# Neoproterozoic glacial origin of the Great Unconformity

**DOI:** 10.1073/pnas.1804350116

**Published:** 2018-12-31

**Authors:** C. Brenhin Keller, Jon M. Husson, Ross N. Mitchell, William F. Bottke, Thomas M. Gernon, Patrick Boehnke, Elizabeth A. Bell, Nicholas L. Swanson-Hysell, Shanan E. Peters

**Affiliations:** ^a^Berkeley Geochronology Center, Berkeley, CA 94709;; ^b^Department of Earth and Planetary Science, University of California, Berkeley, CA 94720;; ^c^School of Earth and Ocean Sciences, University of Victoria, Victoria, BC V8W 2Y2, Canada;; ^d^Department of Applied Geology, Curtin University, Perth, WA 6845, Australia;; ^e^Southwest Research Institute, Boulder, CO 80302;; ^f^Ocean and Earth Science, University of Southampton, Southampton SO17 1BJ, United Kingdom;; ^g^Department of the Geophysical Sciences, The University of Chicago, Chicago, IL 60637;; ^h^Chicago Center for Cosmochemistry, Chicago, IL 60637;; ^i^Department of Earth, Planetary, and Space Sciences, University of California, Los Angeles, Los Angeles, CA 90095;; ^j^Department of Geoscience, University of Wisconsin–Madison, Madison, WI 53706

**Keywords:** Great Unconformity, snowball Earth, glacial erosion, zircon, Cambrian explosion

## Abstract

The Great Unconformity, a profound gap in Earth’s stratigraphic record often evident below the base of the Cambrian system, has remained among the most enigmatic field observations in Earth science for over a century. While long associated directly or indirectly with the occurrence of the earliest complex animal fossils, a conclusive explanation for the formation and global extent of the Great Unconformity has remained elusive. Here we show that the Great Unconformity is associated with a set of large global oxygen and hafnium isotope excursions in magmatic zircon that suggest a late Neoproterozoic crustal erosion and sediment subduction event of unprecedented scale. These excursions, the Great Unconformity, preservational irregularities in the terrestrial bolide impact record, and the first-order pattern of Phanerozoic sedimentation can together be explained by spatially heterogeneous Neoproterozoic glacial erosion totaling a global average of 3–5 vertical kilometers, along with the subsequent thermal and isostatic consequences of this erosion for global continental freeboard.

## Significance

It has long been observed that the sequence of sedimentary rocks deposited in the past half-billion years often sharply overlies older igneous or metamorphic basement at an erosional surface known as the Great Unconformity. We provide evidence that this unconformity may record rapid erosion during Neoproterozoic “snowball Earth” glaciations. We show that the extent of Phanerozoic sedimentation in shallow continental seas can be accurately reproduced by modeling the accommodation space produced by the proposed glacial erosion, underlining the importance of glaciation as a means for lowering erosional base level. These results provide constraints on the sedimentary and geochemical environment in which the first multicellular animals evolved and diversified in the “Cambrian explosion” following the unconformity.

Earth’s sedimentary cover necessarily rests at depth upon igneous or metamorphic crystalline basement. This contact need not be abrupt, since accumulating sediments gradually recrystallize and metamorphose under increasing heat and pressure. Where observed, however, this transition often takes the form of a spatially abrupt and temporally correlated exposure surface known as the Great Unconformity, a lacuna of both time and mass (1–5). While often deeply buried, the Great Unconformity is exposed in areas of relief such as the Grand Canyon of the southwestern United States, where it was first recognized by Powell et al. (1), most dramatically at the sharp nonconformity between the Paleoproterozoic Vishnu Schist and Cambrian Tapeats Sandstone (6). The ubiquity of this pattern—undeformed clastic sediments deposited directly and unconformably atop Precambrian basement—was subsequently recognized by Walcott (2). Observing a dearth of conformable sections spanning the lower boundary of the Cambrian, Walcott proposed a “Lipalian” interval of continental exposure and erosion, which would have restricted any fossil precursors of the Cambrian fauna to the deep ocean basins. Subsequent investigation has revealed a more complete Proterozoic, including fossiliferous strata and conformable boundary sections; yet the observation of a profound and extensive (if discontinuous) pre-Cambrian unconformity remains (refs. 4 and 5 and Dataset S1). Here we attempt to unite disparate evidence including the zircon Hf and O isotope records, the terrestrial bolide impact record, and the record of continental sediment coverage in the context of this widespread unconformity.

## A Discontinuous Global Unconformity

**Fig. 1.**
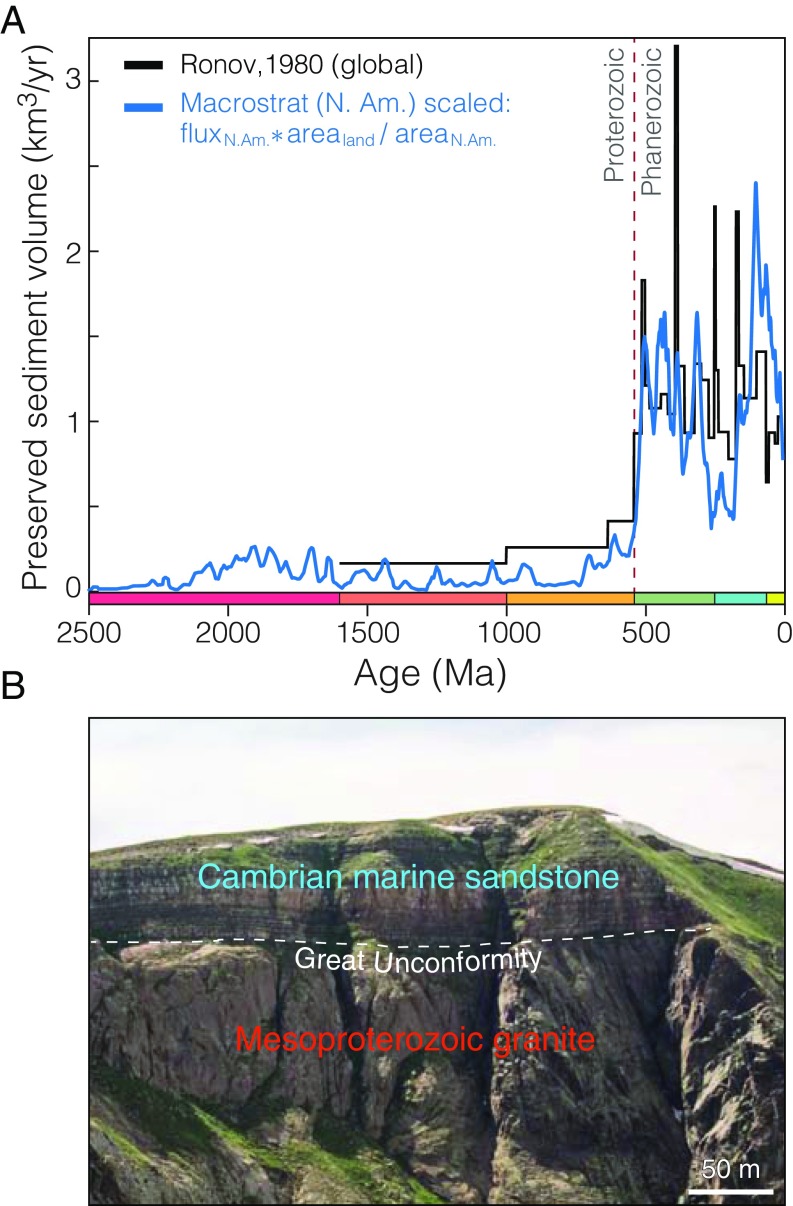
The Great Unconformity. (*A*) Global preserved sedimentary rock volume increases by more than a factor of 5 across the Phanerozoic–Proterozoic boundary in both the estimate of Ronov et al. (4) and a global scaling of North American units from the Macrostrat database by the area ratio of global land area to North American land area (a factor of 6.1) according to Husson and Peters (8), excluding recent alluvium. (*B*) The Cambrian Ignacio quartzite overlies the Mesoproterozoic (∼1.35 Ga) Eolus granite at a sharp peneplanar nonconformity in the Needle Mountains, CO.

The extent and magnitude of secular variation in preserved sediment abundance across the Proterozoic–Phanerozoic boundary were first quantified by Ronov et al. (ref. 4 and Dataset S2), estimating preserved sediment volume flux over the past 1.6 Gy from mapped sedimentary basin areas and stratigraphic thicknesses. The resulting temporal pattern has been subsequently refined in Laurentia by the Macrostrat database (7–9) which (within North America) provides higher-resolution temporal and spatial constraints. Together these records corroborate the presence of a large global shift in preserved continental sediment abundance near the base of the Cambrian (Fig. 1*A* and *SI Appendix*, Figs. S1–S3).

The observed increase from roughly 0.2 ${\text{km}}^{3}$/y of preserved sedimentary rock in the Proterozoic to ∼1 ${\text{km}}^{3}$/y in the Phanerozoic (Fig. 1*A*) might be attributed in principle to either constructive (faster sediment accumulation in the Phanerozoic) or destructive (erosion of Proterozoic strata) processes. However, the abrupt nature of the observed transition presents difficulties for either endmember model. The estimated volume of preserved continental sediment (both in North America and globally) does not follow an exponential abundance curve, as would result from a standard survivorship model (10). Instead, the Proterozoic and Phanerozoic preserved sediment abundance records are individually roughly constant with age—suggesting little influence from erosion on epicratonic marine sediment survival at most times in Earth’s history (7–9). Were the step function in preserved sediment abundance observed in Fig. 1*A* purely a result of concentrated erosion at or near the base of the Cambrian, this would involve the erosion of some 80% of the original Proterozoic sedimentary cover (*SI Appendix*, Fig. S4), totaling as much as 14 vertical kilometers (11).

Alternatively, a purely constructive interpretation would require a roughly fivefold increase in sediment supply and/or continental accommodation space, sustained throughout the Phanerozoic. However, the observed Great Unconformity is profoundly erosional in nature, characteristically juxtaposing fluvial sediment with crystalline basement that was formed at great depth in the crust. For instance, as shown in Fig. 1*B*, the Cambrian Ignacio quartzite is deposited directly upon the Mesoproterozoic Eolus granite (Fig. 1), a pluton with an emplacement depth of approximately 10–15 km (3–4.5 kbar) (12), requiring the erosion of over one-third of the nominal thickness of the continental crust over some subset of the ∼0.9 Gy of geologic history missing from this section.

Posing an additional conundrum in either scenario, the Phanerozoic–Proterozoic boundary is rather unexceptional from a mantle perspective, with no major variation in mantle potential temperature or tectonic style evident in the continental record (13–16). Consequently, it is difficult to conceive of a model where tectonic sediment supply and basin formation increase profoundly as a result of Neoproterozoic solid-Earth processes alone or one in which dramatically increased tectonic exhumation drives unprecedented erosion. Moreover, while the Rodinian supercontinent cycle features a number of noteworthy irregularities—including extroverted supercontinent assembly (17) and an unusual ore deposit profile (18, 19)—it is unclear how such irregularities could contribute to the formation of the Great Unconformity and associated global preserved sediment abundance variations in the absence of significant excursions in mantle potential temperature.

In either a constructive or a destructive endmember scenario, if global sediment supply from tectonic uplift is held constant near Phanerozoic levels, then the depressed Proterozoic sediment volume in Fig. 1*A* suggests that on the order of $10^{9}\;{km}^{3}$ of sediment are absent from the continental crust and deposited instead in the deep ocean basins—either gradually, throughout the Proterozoic due to a diminished sediment storage capacity of the continents in a constructive model, or rapidly during an interval of enhanced erosion near the Proterozoic–Phanerozoic boundary in a destructive model. Indeed, before the plate tectonic revolution, the missing sediments from Walcott’s “Lipalian interval” were generally expected to reside in the ocean basins (2, 20); their absence, along with the young age of the ocean crust, was considered a significant point of evidence in favor of seafloor spreading and plate tectonics (20). In a plate tectonic model, much sediment accumulated on the oceanic crust is consumed by subduction—presently at a rate of about 1.65 ${km}^{3}$/y (21). Due to its low density and fusibility, however, subducted sediment in the mantle wedge is often incorporated into new arc magmas (21, 22); consequently, a chemical or isotopic signature of subducted sediment (if sufficiently voluminous) may be preserved within the igneous record.

## Zircon Hf and O Isotope Systematics

One isotopic system amenable to the detection of such a sediment subduction signature is the radiogenic hafnium isotope system in zircon. In this system, ^176^Hf is produced by the decay of ^176^Lu with a 36-Gy half-life. Since lutetium is more compatible in Earth’s mantle than hafnium, the mantle evolves over time toward more radiogenic Hf isotope compositions (e.g., higher ^176^Hf/^177^Hf) than the crust; this evolution is reported in terms of εHf or parts per 10,000 relative to the isotopic composition of average chondrite (CHUR) (24) at any given time. Notably, the common accessory mineral zircon crystallizes with low Lu/Hf and is readily datable by U-Pb geochronology, permitting the accurate calculation of initial Hf isotopic composition at the time of zircon formation. Due to extremely slow diffusion in the dense zircon crystal lattice, zircons typically retain their closed-system isotopic and elemental composition after crystallization, if not extensively metamict (25). Moreover, zircon is produced most voluminously by felsic magmatism (26) particularly in continental arcs (27). Consequently, the erosion of a sufficiently large mass of felsic crust may be expected to increase both the proportion of sediment-filled trenches and the global rate of sediment subduction, producing a negative Hf isotopic excursion in average global zircon $\varepsilon {Hf}_{i}$, considering the strong correlation between trench sediment thickness and arc zircon εHf observed in more recent zircons (*SI Appendix*, Fig. S8).

**Fig. 2.**
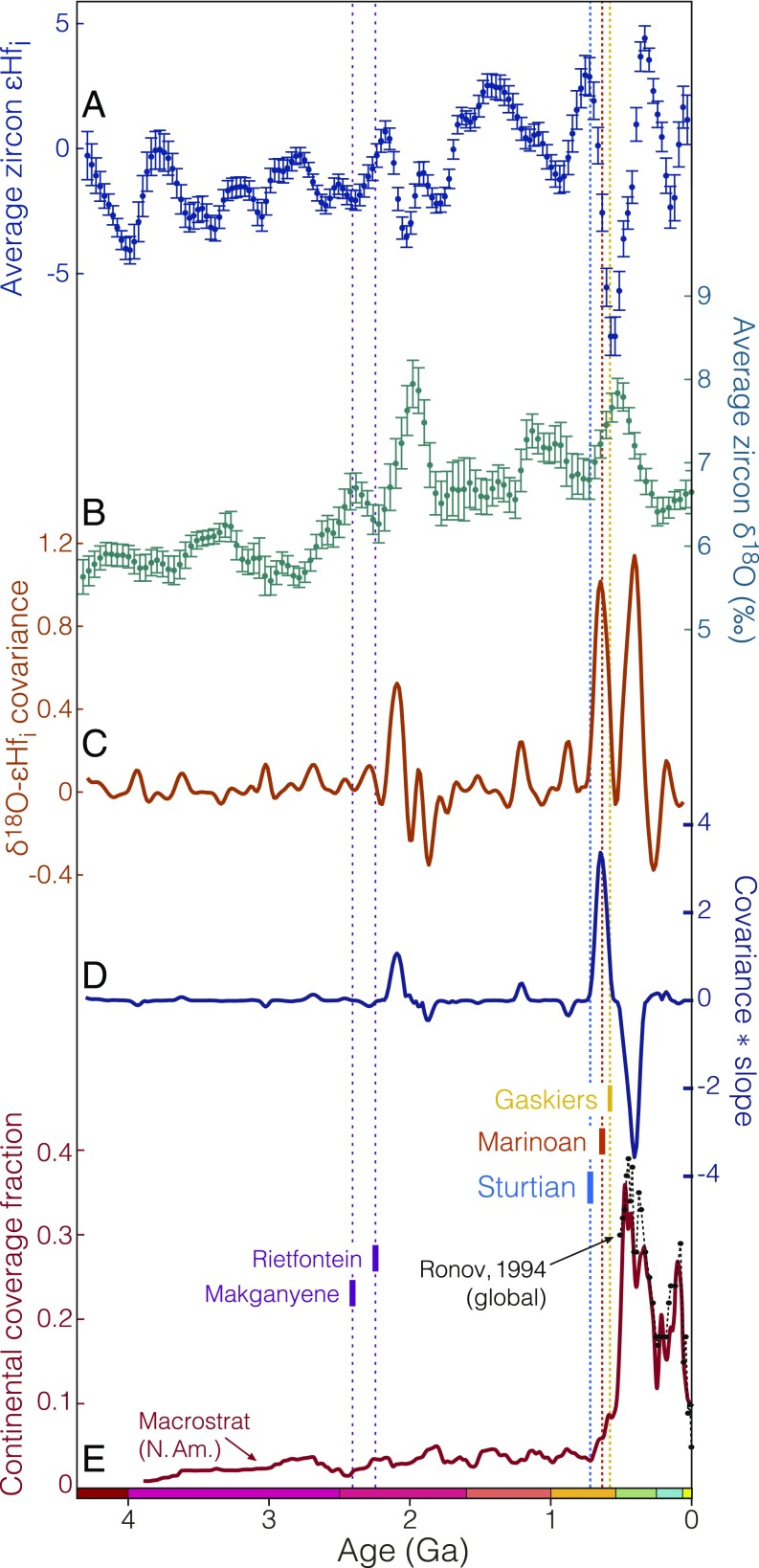
Zircon isotope variability and continental sediment coverage throughout Earth’s history. (*A*) Average zircon εHf. (*B*) Average zircon $\delta^{18}$O. (*C*) The covariance between standardized zircon εHf and $\delta^{18}$O. Positive covariance indicates times where average zircon oxygen and hafnium isotopes both indicate either increasing or decreasing crustal recycling in new magmas. (*D*) The product of standardized εHf - $\delta^{18}$O covariance with standardized average slope. Large positive values indicate high covariance and increasing crustal reworking. Large negative values indicate high covariance and decreasing crustal reworking. (*E*) Fraction of North American continental area covered by marine sediment (age uncertainty represented by σ = 10 My Gaussian kernel) from Macrostrat (7–9), along with the corresponding global Phanerozoic record of Ronov (23).

To quantify crustal average εHf evolution over the past ∼4.4 Gy, we study a dataset of 29,523 zircon U-Pb age and Hf and/or O isotopic analyses using the weighted bootstrap resampling method of Keller and Schoene (13). While sampling and preservation biases are inescapable in the geologic record, this approach accurately propagates uncertainty in age and composition of each sample, while mitigating sampling bias via resampling weights inversely proportional to temporal sample density (13, 16, 28). The result is a continuous record of mean $\varepsilon {Hf}_{i}$ in zircon and 2 SE uncertainty of the mean for 90-My age bins between 0 Ga and 4.35 Ga (Fig. 2*A*).

Average initial zircon εHf remains broadly near zero throughout all of geological history (Fig. 2*A*), close to the isotopic composition of a reservoir with chondritic Lu/Hf. Variations in zircon εHf at the global scale have been traditionally attributed to the supercontinent cycle (29–31). Indeed, moderate fluctuations in this global mean zircon εHf occur throughout Earth’s history on plate tectonic timescales, with significant spectral power at Wilson cycle periods of ∼500–700 My (*SI Appendix*, Fig. S10). However, all other variations are eclipsed in magnitude by a single negative anomaly which begins in the earliest Cryogenian and persists into the Paleozoic, representing by far the most dramatic excursion in the preserved zircon Hf isotope record.

Alone, this Hf isotope anomaly requires the recycling of old, felsic crust. There are many potential mechanisms through which this may occur, but if such remelting is to represent a significant fraction of the global magmatic flux, thermal constraints favor a lower crustal or mantle setting; in this context we consider two endmember scenarios. If recycling were to occur by, e.g., remelting of hot deep crust by basalt pooling near the crust–mantle boundary, the oxygen isotope composition of the resulting partial melt should largely reflect that of the preexisting igneous continental crust. If, however, recycled crust has instead been exposed at or near Earth’s surface, subjected to hydrothermal alteration, or processed through the hydrosphere (as in the case of subducting eroded crust), a positive oxygen isotope anomaly reflecting low-temperature aqueous alteration may coincide with the observed Hf excursion. Fig. 2 reveals just such a correlation; a moving-window covariance estimate confirms the visually evident correlation between the Cryogenian and Ediacaran zircon O and Hf isotope records. In principle, such a correlation is independent of the geologic process by which sediment is recycled into new magmas. However, nonarc magmas produced by sediment melting are a small proportion of global magmatism (Himalayan leucogranites, for instance (32)—but these represent a very small proportion of Cenozoic magmatism, and even here, sedimentary material is transported only to depths and temperatures conducive to anatexis by the subduction and underplating of the Indian continent under Eurasia).

Considering sediment subduction to be the dominant mechanism of recycling sediment into new magmas, as suggested by crustal mass balance (21), a more specific indicator of sediment subduction is provided by the product of the calculated εHf-$\delta^{18}$O covariance with the average slope of the standardized Hf and O isotope records. This product may be considered crudely analogous to the derivative of sediment subduction rate (*Materials and Methods* and *SI Appendix*, Fig. S9), highlighting intervals where both isotope systems indicate consistently increasing (positive product) or decreasing (negative product) recycling of surficially altered felsic crust. The results (Fig. 2*D*) reveal a distinct pairwise anomaly near the time of the Great Unconformity across the Proterozoic–Phanerozoic boundary, with an unprecedented increase in the recycling of continental crust into new magmas in the late Neoproterozoic, followed by a largely Phanerozoic recovery.

While the timing of the observed negative Hf isotope anomaly is potentially consistent with erosion and subduction of crust elided by the Great Unconformity, the required volume of sediment would be large. Using generally conservative estimates for average crust and mantle εHf and continental magmatic flux, we calculate (*Materials and Methods*) that the observed Hf isotope excursion would suggest the recycling of some $2.4\times 10^{8}\;{km}^{3}$ of average crust, corresponding to the erosion of 1.6 km of crust globally if distributed evenly across the continents. Accounting for the low recycling efficiency of subducted Hf into new arc magmas—which is poorly known but likely less than 50%, considering the immobility of Hf in slab fluids (33)—would suggest even larger volumes of subducted crust, ∼3.2 km or greater.

## Neoproterozoic Glaciation and Erosion

Erosional unconformities are common throughout the geologic record and often have a plausible tectonic cause. The same could be said locally for specific exposures of the Great Unconformity (6). However, it is unclear how any local tectonic explanation could produce the observed global variations in preserved sediment abundance (Fig. 1) or crustal recycling (Fig. 2). Neoproterozoic glacial erosion (34) provides a simple mechanism which may reconcile rapid global erosion and sediment subduction with the constraints of the sedimentary record. Glaciers are unique among erosive agents in their ability to alter erosive base level: Glaciation promotes continental denudation both indirectly by lowering global sea level (exposing the continents to subaerial erosion) and directly through subglacial erosion. While rates are variable, in the presence of a large topographic gradient modern subglacial erosion has proved sufficiently erosive to effectively limit global mountain height, evidently outstripping tectonic uplift rates on the order of kilometers per million years (35).

Continental glaciation extended to low paleolatitudes in three well-established Neoproterozoic intervals: the Sturtian (717–660 Ma), Marinoan (641–635 Ma), and Gaskiers (∼580 Ma)—the first two envisioned as global “snowball” events (36, 37) and the Gaskiers as an extensive, but not pan-glacial, event (38). While ice sheet thickness on a snowball Earth is imperfectly constrained and likely heterogeneous (0–6 km) (39–41), glaciation on all continents analogous to that currently found in Antarctica (∼2 km average thickness) would lower sea level by ∼787 m before isostatic adjustment. After isostatic and local gravitational adjustments, modeled freeboard for ice-covered Neoproterozoic continents is variable but positive, with global averages of 400–650 m for each glacial episode (39). Moreover, if not otherwise constrained by air or water temperature, ice base level may extend up to 0.89 km below sea level per kilometer of ice sheet thickness. Such a configuration would provide a large gravitational potential energy gradient to drive erosion, while isostatically permitting more than 12 km of vertical erosion of typical continental crust by a 2-km ice sheet.

The extent of ice-free ocean available to sustain hydrological cycling during such global glaciation is controversial (41, 44). However, precipitation rates driven by sublimation alone appear sufficient for the development of localized wet-based ice streams with high basal sliding velocities and consequent erosive potential (40); evaporation from cryoconite ponds [a notable sink for solar radiation in a snowball state (45)] might further enhance hydrological cycling. Much of the characteristic field evidence for Neoproterozoic glaciation is unmistakably erosional, including striated pavements, striated and exotic clasts and dropstones, and preserved glacial diamictites (36, 46, 47). Although not always well exposed, direct unconformable contact between Neoproterozoic glacial sediments and Archean to Neoproterozoic crystalline basement may be found on most continents (48).

While the Great Unconformity surface in Fig. 1*B* allows some ∼0.9 Gy for exhumation of crystalline basement to the surface, other sections may be found where a basement unconformity directly superposes Neoproterozoic glacial diamictites with crystalline basement only some tens to hundreds of million years older. In the Mirbat region of Oman, for instance, Sturtian glacial diamictites and syn-glacial sediments unconformably overlie a juvenile crystalline basement complex with ages ranging from ∼810 Ma to as young as 696.7 ± 0.5 Ma (49–51), raising the possibility of exhumation of syn-Sturtian phaneritic igneous rocks to the surface during the glacial episode. In sections with less exceptional preservation, juvenile clasts in Neoproterozoic diamict may provide additional evidence for direct glacial erosion of young crystalline basement: For instance, Sturtian glacial deposits of the Rapitan Group contain granitic basement clasts as young as 755 ± 18 Ma (52). Since exploitation of a gravitational potential energy gradient facilitates rapid glacial erosion (35), glacial erosion of young basement may be concentrated in areas of preexisting topography. Critically, Neoproterozoic glacial erosion need not be spatially uniform to produce the observed sediment subduction signature—nor should we expect uniform glacial erosion considering the negligible erosional potential of cold-based ice, the localized erosion of outlet ice streams, and the preservation (often in areas of tectonic subsidence) of relatively complete sections lacking appreciable glacial erosion (e.g., ref. 53).

Modern glacial erosion rates are highly variable, estimated to span some four orders of magnitude from ∼0.01 mm/y to ∼100 mm/y (54). For comparison, 4 km of erosion over 64 Ma of Neoproterozoic glaciation would require an average erosion rate of only 0.0625 mm/y—nearly two orders of magnitude slower than recent direct estimates for the modern Greenland ice sheet (55); while some such estimates (if reversible processes are involved) must be corrected for timescale dependence, the required rate is nonetheless well within the range of physical feasibility for glacial erosion. Moreover, while Sturtian and Marinoan glacial deposits evidence accumulation rates 3–10 times slower than modern equivalents (45, 56), accommodation space—not depositional process or sediment supply—is likely the rate-limiting variable at applicable (>5 My) timescales (56); in the absence of such accommodation, sediment will not accumulate on the continents, but rather in the ocean basins below erosional base level. Consistent with an accommodation-limited model, Neoproterozoic diamictites may reach kilometer-scale thicknesses where directly accommodated by local syndepositional tectonism (46, 47). In the context of global glaciation, accommodation must be considered as a competition between subsidence and regional upland erosion: Local thermal or tectonic subsidence may be thwarted by isostatic rebound from regional erosion (*SI Appendix*, Fig. S11).

Delivery of eroded sediment to the deep ocean basins is a critical requirement for the production of the observed Great Unconformity (where much of the eroded crust is not found elsewhere on the continents) and is consistent with predictions for Neoproterozoic glacial erosion. During pan-glacial conditions, the locus of deposition should shift to deeper waters as a result of (*i*) lowered erosional base level; (*ii*) direct transport of eroded sediment by erosive outlet glaciers [such as those responsible for the Chuos paleovalley (47)], which in the present day are often associated with overdeepened fjords that extend to the edge of the continental shelf; and (*iii*) settling of fine glacial flour in deep ocean basins. In more simplistic terms, when all continental area is below ice base level during a snowball glaciation, most sediment is transported entirely off the continental shelves and into the ocean basins, where it is ultimately subducted—just as suggested by the observed Hf and O isotope records (Fig. 2).

Direct and indirect implications are widespread when considering a geological event as nonuniformitarian as the proposed kilometer-scale Cryogenian erosion, resulting in numerous testable predictions. For instance, crust exhumed by large-scale erosion cools as thermal diffusion adjusts to the new relative position of the surficial boundary condition. A range of existing thermochronologic inversions, although geographically variable, appear permissively consistent with ∼100–300 °C (∼3–9 km at a 33 °C/km geothermal gradient) of potentially rapid Neoproterozoic crustal exhumation (57–60). Further analyses are required to conduct a systematic global survey of the long-term thermal history of the continents, since a large proportion of existing thermochronologic data is focused on areas of more recent tectonic activity that are unlikely to preserve a record of Neoproterozoic exhumation.

**Fig. 3.**
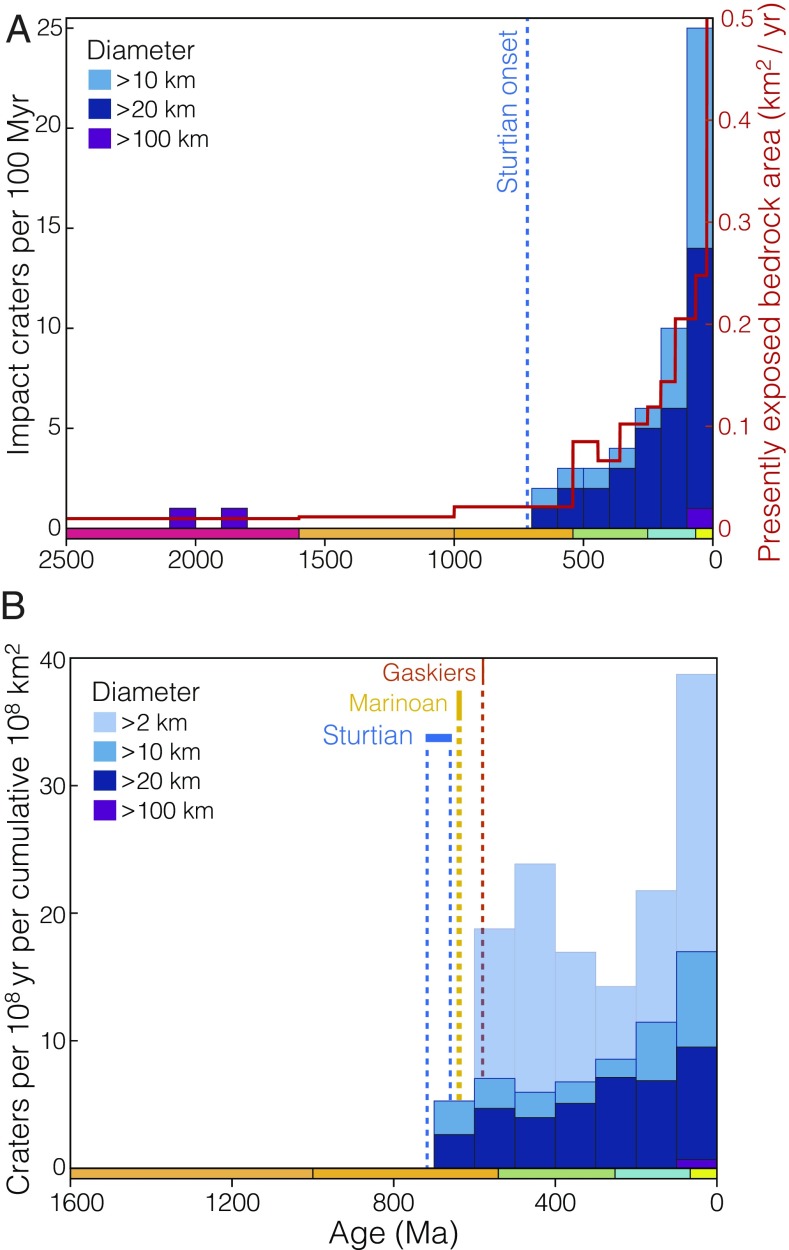
The record of impact craters preserved in Earth’s continental crust with formation ages known to within ±75 My (1-σ) from the PASSC database (42). (*A*) Absolute crater counts (left axis) for several size ranges tallied in 100-My bins over the past 2.5 Ga, plotted alongside global exposed bedrock area in ${km}^{2}$/y (right axis) (43). (*B*) Apparent impact cratering rate per unit bedrock area area tallied in 100-My bins for crater diameters from 2 km to >100 km.

One specific testable prediction concerns the terrestrial bolide impact record: Impact craters are surficial features, subject to destruction by exhumation and erosion. Since impact craters are shallow relative to their diameter, kilometer-scale Neoproterozoic erosion, if widespread, should significantly reduce the preservation potential of all but the largest impact craters. Fig 3*A* shows the record of known terrestrial impact craters larger than 10 km diameter with ages known within ±75 My, updated from the Planetary and Space Science Center (PASSC) compilation (42). While the abundance of >10-km impact craters closely follows exposed bedrock area for the past 700 My, only two craters matching the criteria of Fig. 3 predate the onset of Sturtian glaciation, both deeply eroded remnants of massive craters: Sudbury and Vredefort, eroded to depths of 4.2–5.8 km and 8–11 km, respectively (61, 62). This trend is particularly striking when considered as a function of crater density per unit area (Fig. 3*A* and *SI Appendix*, Fig. S12), with an abrupt truncation of <100-km diameter craters before 700 Ma and <10-km diameter craters before 600 Ma—temporally consistent with progressive Neoproterozoic glacial erosion.

More qualitatively, we may extend our analysis of preservational bias from the bolide impact record to consider a wide range of geological features with an affinity for the shallow crust. For instance, we may predict that any mineral assemblage which cannot survive prolonged low-grade metamorphism in a normal continental geotherm should be less abundant before the Sturtian. This prediction appears consistent with the noted absence of thermodynamically fragile (U)HP/LT assemblages such as jadeitites and glaucophane eclogites before ∼700 Ma (63, 64), although not uniquely so (65, 66). The same prediction appears likewise consistent with the strong (and apparently stepwise) “preservational bias toward [mineral] deposits of the Phanerozoic Eon” reported by Liu et al. (page 2 in ref. 19).

## Consequences of Rapid Crustal Erosion

The timing of Neoproterozoic glaciation is remarkably consistent with both the observed zircon isotopic excursions and continental sediment coverage history at the scale of Fig. 2. This discontinuous record is an expected consequence of the stepwise preservation potential imposed by focusing extensive, if nonuniform, kilometer-scale continental denudation into a few discrete episodes of intense glacial erosion amid a background of comparatively negligible (<2.5 m/My) cratonic exhumation (67). Consequently, the observed sediment coverage record may be considered in part a discretization of the exponential survivorship curve (10) that would result from continuous erosion (e.g., *SI Appendix*, Fig. S4).

In this discretization, each glacial epoch acts as a filter in the crustal record, removing some proportion of older sediments via erosion. Since erosional surfaces are subject to capture by subsequent erosion, the most dramatic unconformity (and largest step in preserved sediment abundance) may be inherited by the most recent glaciation, consistent with Fig. 2*E*. However, such erosion does not preclude a constructive contribution to the Great Unconformity; to the contrary, it requires one. Continental thinning through erosion directly decreases continental freeboard, raising relative sea level and providing accommodation space for sediment accumulation. While this new accommodation space may be temporarily moderated by thermal buoyancy given erosional advection of the continental geotherm, continental erosion nonetheless inevitably leads to increased continental sediment storage, as proposed by ref. 8.

**Fig. 4.**
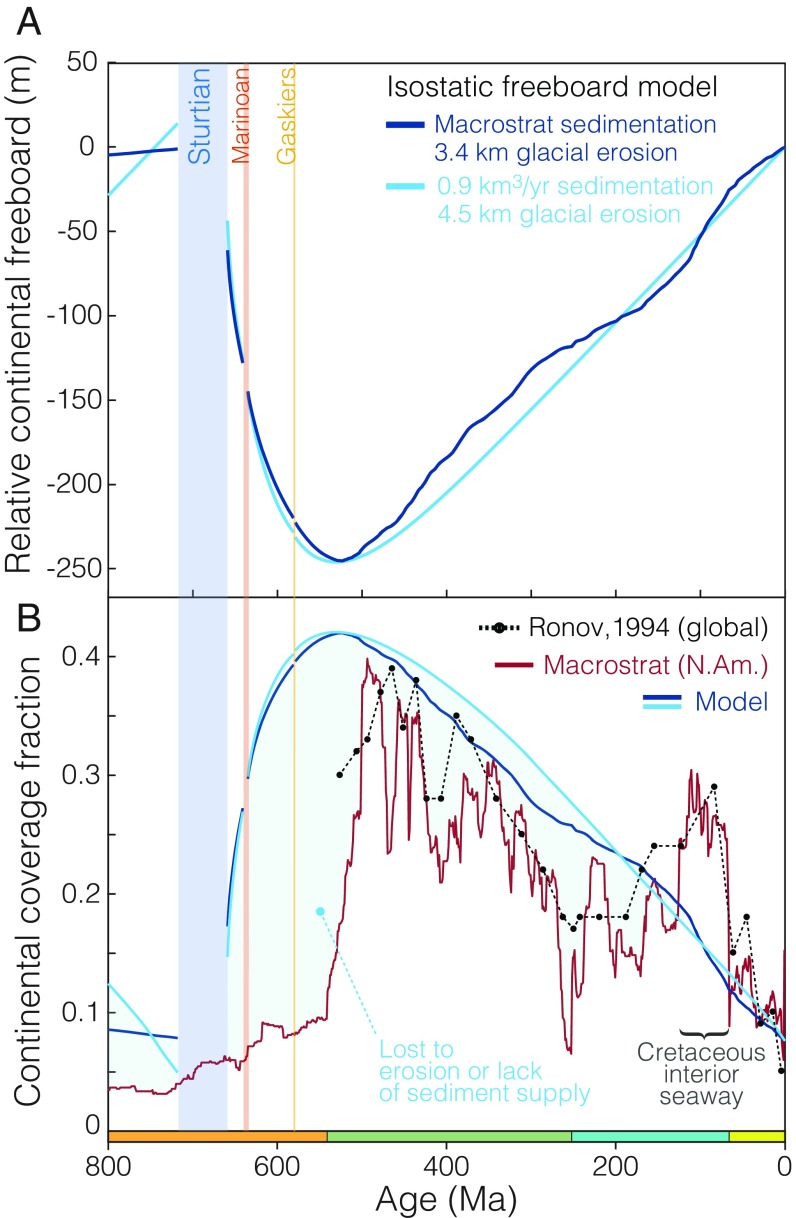
Isostatic global sea level and continental coverage model. (*A*) Temporal evolution in average continental freeboard driven by erosion, subsequent thermal subsidence, and sediment accumulation. Neoproterozoic glacial erosion is distributed in proportion to the duration of each glacial interval. (*B*) Corresponding modeled continental coverage fraction assuming a constant hypsometric profile, compared with the observed North American record from Macrostrat (7–9) and Ronov’s (23) global record of Phanerozoic marine sediment coverage.

To quantify the depositional consequences of rapid Neoproterozoic erosion, we constructed a 1D model of continental freeboard, combining the effects of erosion, isostasy, thermal subsidence, and sediment accumulation over the past 800 My. Using either the Phanerozoic net sedimentation rate from Fig. 1*A* or a constant assumed rate of 0.9 ${km}^{3}$/y, varying the model magnitude of Neoproterozoic erosion directly influences initial freeboard via mass balance (*SI Appendix*, Fig. S13). Near-modern freeboard at 750 Ma is reproduced with 3.4–4.5 km Neoproterozoic glacial erosion, producing in each case a nearly 250-m isostatic excursion in relative sea level (Fig. 4*A*). Using a modern hypsometric profile (*SI Appendix*, Fig. S15) to convert from sea level to continental submergence fraction as illustrated in Fig. 4*B*, this 250-m excursion corresponds remarkably well with the observed macrostratigraphic record of marine sediment coverage.

The first-order success of this 1D freeboard model prediction is particularly remarkable considering that the model includes no consideration of local tectonics. However, one feature remains problematic: the time delay between the end of Neoproterozoic glaciation and the Cambrian increase in preserved sediment abundance. Potential causes for this misfit may fall into three broad categories:

*i*) Erosional loss of the Ediacaran record, glacial or otherwise, provides the most direct mechanism. Maintaining low preserved sediment abundance over the 92 My of the Ediacaran (and particularly the 39 My from the Gaskiers to the base of the Cambrian) through erosional means would be trivial compared with the kilometer-scale erosion we propose for the Cryogenian. While a late Ediacaran glaciation has been suggested (68, 69), precise geochronological constraints are lacking and key observations [e.g., *Cloudina* in the matrix of an Ediacaran diamictite (70)] have not been replicated.
*ii*) Nondeposition resulting from sediment starvation may be expected if glacial peneplanation (71) sufficiently reduces the available topography; reduced sediment supply could persist on tectonic timescales until orogenesis provides a renewed clastic input. However, there are Ediacaran basins which are not sediment starved.
*iii*) Chronological bias may result in an underestimation in the volume and extent of Ediacaran sediments if ambiguous units are mistakenly assigned to the Cambrian. The residual currency of the phrase “Precambrian basement” testifies to the historical association of the first sediments above crystalline basement to the Cambrian system. However, we hope that this known problem (72) has been largely corrected over recent decades.

While none of the above hypotheses alone is entirely satisfactory, all imply a range of testable predictions that may be better understood with future work.

## Inferences and Conclusions

The first quantification of continental submergence by Egyed (76) indicated dramatic emergence throughout the Phanerozoic (i.e., declining marine coverage), as in Fig. 4*B*. While the original interpretation of this record has been obviated by plate tectonics (77), the paradigm of monotonic continental emergence as a result of global cooling has persisted (78, 79). We suggest that this paradigm must be reevaluated. The correspondence between the modal elevation of the continents and global sea level (*SI Appendix*, Fig. S15) is not coincidental, but rather a direct consequence of subaerial erosion on a tectonically active Earth (80); given active orogenesis and felsic continental crust, any buoyant continental mass with negative freeboard must thicken by orogenesis and sedimentation until it reaches zero or slightly positive average freeboard, if not otherwise limited by delamination or gravitational collapse. The negative continental freeboard which enabled extensive continental coverage and subsequent recovery (i.e., emergence) throughout the Phanerozoic (9, 76) may thus be an anomaly enabled by glacial erosion below ice-free oceanic base level.

**Fig. 5.**
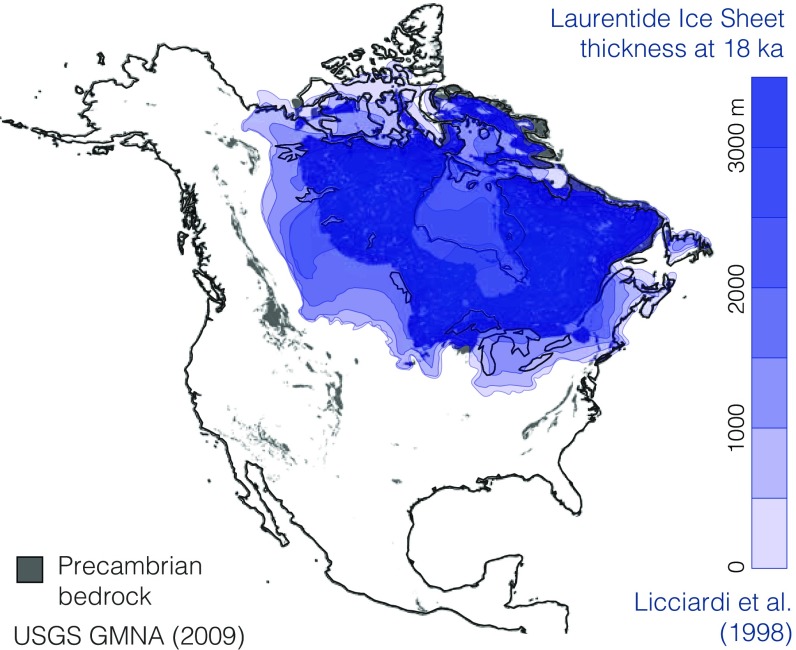
Capture of the Great Unconformity by Laurentide glacial erosion, illustrated by the correspondence (73) between Precambrian basement exposure as mapped in the Geologic Map of North America (74) and the extent of the Laurentide ice sheet at 18 ka as estimated by Licciardi et al. (75). Note the survival of Phanerozoic cover under the ice divide near Hudson’s Bay, where basal sliding velocities are low.

While nonconformity between sediment and crystalline basement is ubiquitous on all continents, it is highly diachronous (6). This diachroneity of amalgamated unconformities has helped to obscure the global significance of Neoproterozoic glacial erosion. Proterozoic or Archean basement is commonly exposed at the surface even today (Fig. 5)—an ongoing Great Unconformity. However, exhumation at such sites likely results from multiple ancient (e.g., Neoproterozoic) unconformities collapsed, captured, and deepened by more recent erosion. A remarkable correspondence has been noted between Precambrian bedrock exposure and glaciation (Fig. 5); virtually all nonorogenic exposures of Precambrian basement have been subject to glaciation during either the Late Paleozoic Ice Age or the Quaternary (73, 81) (*SI Appendix*, Fig. S16). In this context, we suggest that the present icehouse epoch may display comparatively high continental erosion rates (82) relative to the Phanerozoic background, reconciling unsustainable modern erosion rates of 0.05–0.5 mm/y (i.e., 50–500 km/Gy) with the survival of Archean crust and lithosphere (67).

Considering the glacigenic model for the Great Unconformity proposed here, zircon Hf and O isotopes may represent the first paleoerosion proxy preserved in Earth’s igneous record, preserving a signal of surface earth processes over billion-year timescales. In this context, we note that a set of smaller but correlated Paleoproterozoic excursions in the zircon Hf and O isotope records circa 2.2 Ga appears following a known period of Paleoproterozoic glaciation (83). Given the lack of geologic evidence for glacial deposits between the ∼2.2-Ga Rietfontein (83) and ∼0.72-Ga Sturtian (37) glaciations, Earth may have experienced a prolonged period of weathering and regolith development (84) with comparatively little marine sediment accumulation on the continents due to a lack of glaciation-derived accommodation space. Thus, Neoproterozoic global glaciation may have been responsible for initiating a Phanerozoic cycle of continental sedimentation with enhanced Paleozoic continental inundation and sediment accumulation relative to the preceding late Proterozoic. We conclude that the Phanerozoic sedimentary record is best explained by a Great Unconformity of inherently coupled erosive and constructive genesis, with Neoproterozoic glacial erosion governing the subsequent history of continental freeboard and sediment accumulation (Fig. 4*B*). As such, the environmental and geochemical changes that led to the diversification of multicellular animals (5) may be considered a direct consequence of Neoproterozoic glaciation.

## Materials and Methods

To investigate anomalies in the continental rock record near the Proterozoic–Phanerozoic boundary, we assemble a range of stratigraphic, geochemical, and geological datasets. Stratigraphic data for North America are obtained from the Macrostrat database (macrostrat.org), originally produced by Peters (7) by digitization of the the American Association of Petroleum Geologists Correlation of Stratigraphic Units of North America (COSUNA) charts (85). This stratigraphic record of the Great Unconformity is interpreted alongside compiled zircon Hf and O isotope geochemistry, as well as terrestrial and lunar bolide impact datasets. Finally, stratigraphic and geochemical results are integrated and interpreted in the context of an isostatic and thermal model of continental freeboard. Computational source code and data are freely available at https://github.com/brenhinkeller/GreatUnconformity.

### Zircon Isotope Systematics and Monte Carlo Analysis.

We compiled zircon Hf and O isotopic compositions along with U-Pb ages for igneous and detrital zircons from the preexisting datasets of Belousova et al. (86), Dhuime et al. (87), and Spencer et al. (88)/Payne et al. (89), augmented by some further compilation of literature data, resulting in a dataset of 35,368 analyses from all continents (*SI Appendix*, Figs. S5 and S6), of which 29,523 are unique. To obtain a maximally representative temporal record of zircon Hf and O isotopic composition, we applied weighted-bootstrap resampling following the approach of Keller and Schoene (13, 16). While ages are known directly for each analysis, geographic locations are largely absent from the dataset. Consequently, sample weights $w_{i}$ for each sample i are assigned inversely proportional to temporal sample density following the relation

$$w_{i}=1/\overset{n}{\underset{j=1}{\sum }}\frac{1}{{\left(t_{i}-t_{j}\right)}^{2}+1},$$

where n is the number of samples in the dataset and t is sample age. Subsequently, the dataset is resampled with replacement, with sampling probability proportional to sample weight. This weighting produces a more even temporal distribution (*SI Appendix*, Fig. S7) and obviates the manual elimination of, e.g., duplicate analyses. Throughout resampling, each geochemical measurement (e.g., a single zircon Hf isotope ratio) is represented as a Gaussian random variable with a known mean and SD such that a new value is drawn from this distribution each time the dataset is resampled, thereby fully representing analytical uncertainty. Average results throughout Earth’s history are presented as an average and 2 SE of the mean for overlapping 90-Ma windows between 0 Ma and 4,350 Ma (e.g., Fig. 2).

The global average zircon Hf and O isotope timeseries both record the recycling of preexisting crust into new magmas. Positive O isotope excursions above the mantle baseline (∼5.5/mil) reflect the recycling of silicate crust that has undergone low-temperature aqueous alteration at Earth’s surface (i.e., sediment), while negative Hf isotope excursions reflect the recycling of old, felsic crust that has undergone less ^176^Hf ingrowth than the convecting mantle. Zircon Hf and O isotope averages vary throughout the supercontinent cycle as the proportion and preservation of arc, rift, and collisional magmatism vary (29–31); such normal variations are observed throughout the entirety of the preserved record, with roughly the expected periodicity (*SI Appendix*, Fig. S10). Compared with this normal tectonic background, the Neoproterozoic excursions are notable both in magnitude and in the covariance between Hf and O isotope records. While atypical O and Hf isotope characteristics of Neoproterozoic zircon have been previously noted (30, 31, 90), their systematic global covariance and the broader implications thereof have not been previously explored.

To assess the importance of sediment subduction, we examined the covariance between the zircon Hf isotope signature of felsic crustal recycling and the zircon O isotope signature of sediment recycling, following a procedure illustrated in *SI Appendix*, Fig. S9. First, to remove any scale dependence or extraneous covariance from long-term secular crustal evolution (as opposed to distinct crustal recycling episodes), both isotopic records are detrended and normalized to unit variance, with the εHf isotopic signal inverted such that increasing recycling is positive for both systems (*SI Appendix*, Fig. S9 *A* and *B*). The resulting covariance is illustrated in *SI Appendix*, Fig. S9*C*. This raw covariance is positive where the Hf and O signals either increase or decrease in concert: Both the excursion and recovery of the Neoproterozoic isotope anomaly yield large positive covariance peaks. Since we wish to distinguish between excursion (increasing sediment subduction) and recovery (decreasing sediment subduction back to baseline), we additionally examine the product of this covariance with the average slope of the two Hf and O isotope signals (*SI Appendix*, Fig. S9*D*). Since the average slope tends to zero in the case of negative covariance, the covariance–slope product (*SI Appendix*, Fig. S9*E*) emphasizes large positive covariance co-occurring with either increasing or decreasing sediment subduction; individual subduction events thus appear as characteristic pairwise features with a positive excursion peak immediately followed by a negative recovery peak. Two such events are evident: a Paleoproterozoic pair with an excursion beginning circa 2,200 Ma and a much larger Neoproterozoic pair with an initial excursion coincident with the onset of the Sturtian glaciation [∼717 Ma (37, 91)], a nadir at ∼560 Ma, and an ∼220-My recovery that is complete by ∼340 Ma. Notably, the essentially immediate (on gigayear scales) onset of the excursion following Sturtian glaciation is consistent with the fast recycling of sediment into new magmas (<7–9 Ma from erosion to eruption) suggested by cosmogenic 10Be anomalies in modern arc magmas (92)—while the timescale of recovery is entirely consistent with the ∼200-My characteristic timescale for complete turnover of the oceanic crust (and thus complete subduction of any accumulated sediments into the ocean basins).

Given the observed magnitude of the global Hf isotope excursion, we may estimate the minimum required volume of subducted crust. Taking the compiled zircon εHf datastet as an estimate of average εHf of new igneous crust throughout Earth’s history, we may calculate the average crustal εHf at any subsequent time accounting for Hf ingrowth in accordance with Lu/Hf ratios for each whole-rock sample in the dataset of Keller and Schoene (13), obtaining Neoproterozoic values ranging from −33.7 ε at 717 Ma to −34.9 ε at 635 Ma. Since a more negative crustal endmember will result in lower estimated volume of subducted crust, we choose −35ε as a minimum value. This estimate is conservative since the zircon record samples only zircon-bearing magmas, which are predominantly felsic (26) and may exhibit more negative initial εHf than average crust due to a greater contribution from assimilation of preexisting crust than, e.g., a primitive basalt. Meanwhile, as the most positive reservoir in εHf space, the evolution of the depleted mantle may be traced as the upper limit of the compiled εHf field through time, estimating a value of +14ε for the Neoproterozoic (*SI Appendix*, Fig. S6). As seen in Fig. 2, the Neoproterozoic negative εHf excursion ranges from a baseline of +4 ε to a nadir of −8 ε, with a depth of 12 ε, with an average depth of 5.7 ε over the 400-My duration of the excursion. This average depth corresponds to 5.7/(14 − (−35)) = 0.12 of the total range between average crust and depleted mantle, equivalent to shifting 12% of total continental magmatism over the duration of the excursion from a mantle source to a crustal source.

Phanerozoic estimates of rates of volcanic and plutonic magmatism in the continental crust suggest 3–8 ${km}^{3}$/y of arc volcanism and plutonism along with 0.2–1.5 ${km}^{3}$ of intraplate continental magmatism (93). More recent mass balance constraints suggest at least 3.8 ${km}^{3}$/y of arc magmatism is required to avoid long-term crustal destruction. Consequently, we take 5 ${km}^{3}$/y as a relatively conservative estimate of total continental magmatism. In this case, shifting 12% of continental magmatism from a mantle source to a crustal source over 400 My would require the recycling of some $2.4\times 10^{8}\;{km}^{3}$ of average crust. Such a volume corresponds to 1.61 vertical kilometers if distributed evenly over the $1.489\times 10^{8}\;{km}^{2}$ area of the continents. Considering that only a fraction of subducted Hf makes its way into new magmas (depletion of high-field-strength elements such as Hf is a characteristic signature of arc magmatism due to the immobility of these elements in aqueous slab fluids) (33), the true value is likely at least twice that, or $∼5\times 10^{8}\;{km}^{3}$ if this recycling occurs via sediment subduction.

### The Terrestrial Bolide Impact Record.

To obtain an independent constraint on the timing and magnitude of Neoproterozoic erosion, we have examined the terrestrial impact crater record as compiled in the PASSC Earth Impact Database (42), with age constraints updated where applicable. Since bolide impact craters necessarily occur at Earth’s surface, their resistance to erosion is a function of crater depth. Hypervelocity impact craters are characteristically shallow features, with an initial depth around 1/10th of the their original diameter or less (94, 95), decreasing above 15 km diameter such that a Lunar impact crater of 100 km diameter may be only 4 km deep (95). Consequently, all but the largest terrestrial impact craters should be susceptible to erasure by Neoproterozoic glacial erosion. If the Neoproterozoic glacial erosion hypothesis is correct, we expect a dramatic decrease in impact crater preservation potential across the Cryogenian for all but the largest class of terrestrial impacts. While this prediction is broadly confirmed by the raw impact record alone (Fig. 3*A*), the signal of preservation is better resolved by normalizing the impact record to the continental area that was available for impact cratering at some time in the past and is now again exposed at the surface.

We explore two such normalizations, (*i*) to the cumulative area of crust exposed today that is older than a given impact age and (*ii*) to the surface area of crust exposed today of the same age as a given impact crater (*SI Appendix*, Fig. S12 *A* and *B*). The first normalization (by cumulative exposed area, as seen in Fig. 3*B* and *SI Appendix*, Fig. S12*D*) is the most conservative in that the presently exposed area bedrock of age X Ma or greater is the maximum exposed surface area that could preserve an impact of age X Ma. This is a maximum extent because, for instance, 1-Ga bedrock may be extant at 0.5 Ga, but deeply buried and thus unable to record an impact at that time.

The latter normalization (by the relative area of exposed crust of the same age as a given impact, within some binning resolution) is more aggressive but may be considered more natural for sedimentary or volcanic bedrock, which must have been exposed at the time of deposition and thus would have been available as a target for bolide impacts at that time. This normalization results in an even more dramatic discontinuity in preserved cratering rate across the Cryogenian (*SI Appendix*, Fig. S12*C*). The true preservation signal is likely intermediate between *SI Appendix*, Fig. S12 *C* and *D*, but in either scenario strikingly lower preservation potential is suggested for impact craters predating Neoproterozoic glaciation.

### Continental Freeboard and the Sedimentary Record.

The Great Unconformity is manifest in the macrostratigraphic record of continental sedimentation in the form of a series of approximately stepwise increases in preserved sediment abundance between approximately ∼720 Ma and ∼500 Ma (Figs. 2*E* and 4). In an erosional context, each step may be considered to reflect a decreasing probability of any preexisting sediment having survived past a given glacial episode. For instance, sediments older than the Gaskiers may have survived only one Neoproterozoic glaciation, while sediments older than the Sturtian must have survived all three. Moreover, since erosive glaciation tends to capture the evidence of previous erosion, the largest abundance step (and most dramatic unconformity) may be inherited by the most recent glaciation, consistent with the results of Fig. 2*E*. For instance, if the Sturtian and Marinoan together were to erode 3 km of crust, followed by 100 m of sedimentation between 635 Ma and 580 Ma, the Gaskiers need only erode 100 m of sediment to capture the entire (now) 3.1-km unconformity.

To quantify the consequences of Neoproterozoic erosion for continental freeboard and sediment accumulation, we constructed a 1D thermal and isostatic model of the continental crust and lithosphere. On approximately gigayear simulation timescales, isostatic adjustment is assumed to be effectively instantaneous, with postglacial viscous mantle rebound (45, 96) likely complete within a single-million-year model timestep. However, the thermal consequences of kilometer-scale erosion may be more protracted. To account for thermal subsidence as the advected geotherm decays back into equilibrium, along with the direct isostatic effects of erosion and sedimentation, our model assumes a coefficient of thermal expansion of $3\times 10^{-5}$/K, a thermal diffusivity of 1 ${mm}^{2}$/s, an average crustal thickness of 33 km, an average density of 2,818 kg/$m^{3}$ for the continental crust (97), a mantle density of 3,300 kg/$m^{3}$, and a slightly buoyant mantle lithosphere (3,250 kg/$m^{3}$) of 100-km thickness, for a total lithospheric thickness (crust + mantle lithosphere) of 133 km, generally intermediate between expected thermal (98) and elastic (96) lithospheric thicknesses. This model was then perturbed by various scenarios of erosion and sedimentation, with several kilometers of Neoproterozoic erosion followed by either continuous (0.9 ${km}^{3}$/y) or variable (Macrostrat derived, as in Fig. 1*A*) sedimentation rate. For the purposes of Fig. 4 and *SI Appendix*, Fig. S13, the total volume of glacial erosion was partitioned between the three Neoproterozoic glacial intervals in proportion to their duration. However, instead equally distributing erosion between all three glaciations has little impact on the results (*SI Appendix*, Fig. S14).

To better understand the implications of this model for continental emergence and sedimentation, the resulting freeboard curve was translated into expected continental coverage extent, using a present-day hypsometric curve (*SI Appendix*, Fig. S15). The assumption of present-day hypsometry is notably imperfect, but presently unavoidable given an absence of independent constraints on past global hypsometry. Glaciation may significantly alter continental hypsometry—with the potential to either produce or destroy topographic contrast under different conditions (71). Consequently, the global hypsometric gradient is poorly constrained both before and in the immediate aftermath of Neoproterozoic glaciation. The assumption of near-modern hypsometry is more supportable closer to the present day (i.e., the past 500 My), which is perhaps unsurprisingly where model misfit is lower.

As illustrated in Fig. 4, the model results are remarkably consistent with the observed continental coverage extent curve, with continental coverage increasing dramatically in the aftermath of Neoproterozoic erosion and then slowly declining to background as continued sedimentation fills the available accommodation space. While the general agreement between model and observed coverage trends is quite good given the wide range of uncertainties involved, two particular intervals of misfit are apparent: (*i*) a period in the middle Cretaceous where observed coverage substantially exceeds model expectations and (*ii*) systematically lower than expected coverage before ∼500 Ma.

A wide range of factors may introduce such misfit. First, no specific tectonic or orogenic events are included in our simple 1D model. In this context, the relatively low misfit after ∼500 Ma is arguably surprising and suggests that the global rates of relevant local processes such as orogenesis and basin formation may not have varied wildly over the past 500 My. Systematic variation in mantle heat flow may change oceanic spreading rate (99) and midocean ridge height, thus changing average global sea level. Additional misfit may be introduced by erosional or nondepositional unconformity in the record subsequent to the initial Great Unconformity; continental emergence will be overestimated if we are missing the sediments by which we estimate coverage. Any change in the form of the terrestrial hypsometric profile between 800 Ma and today—likely, but difficult to test—would introduce error into the function mapping between continental freeboard and coverage extent. Finally, the accuracy of the observed coverage record is entirely dependent on the accuracy of the underlying geochronological constraints.

One might at first consider this Cretaceous anomaly as a regional bias reflecting the well-known Cretaceous Interior Seaway of North America (100, 101) attributable to, e.g., regional tectonics or dynamic topography. However, the Cretaceous has long been known as a time of anomalous flooding on multiple continents (102), and indeed a positive coverage anomaly is observed even in the coarser-timescale global record of Ronov (23) as seen in Fig. 4*B*. Consequently, this excursion may be more consistent with a global increase in midocean ridge elevation and spreading rate. While controversial, increased mantle heat flow in the Cretaceous has been proposed in conjunction with the Cretaceous Long Normal Superchron and the Kerguelen and Ontong–Java oceanic flood basalt plateau (103–105), potentially consistent with high average ocean ridge elevation and increased sea level for much of the Cretaceous.

### Code and Data Availability.

Macrostratigraphic data are accessible via https://macrostrat.org/api. All compiled datasets and computational source code are available at https://github.com/brenhinkeller/GreatUnconformity.

## Supplementary Material

Supplementary File

Supplementary File

Supplementary File

## References

[1] Powell JW, Thompson AH, Coues E, Goode GB (1875). Exploration of the Colorado River of the West and its Tributaries.

[2] Walcott CD (1914).

[3] Sloss LL (1963). Sequences in the cratonic interior of North America. Geol Soc Am Bull.

[4] Ronov AB, Khain VE, Balukhovsky AN, Seslavinsky KB (1980). Quantitative analysis of Phanerozoic sedimentation. Sediment Geol.

[5] Peters SE, Gaines RR (2012). Formation of the ‘Great Unconformity’ as a trigger for the Cambrian explosion. Nature.

[6] Karlstrom KE, Timmons JM (2012).

[7] Peters SE (2006). Macrostratigraphy of North America. J Geol.

[8] Husson JM, Peters SE (2017). Atmospheric oxygenation driven by unsteady growth of the continental sedimentary reservoir. Earth Planet Sci Lett.

[9] Peters SE, Husson JM (2017). Sediment cycling on continental and oceanic crust. Geology.

[10] Gregor B (1970). Denudation of the continents. Nature.

[11] Husson JM, Peters SE (2018). Nature of the sedimentary rock record and its implications for Earth system evolution. Emerging Top Life Sci.

[12] Hunter RA, Andronicos CL (2012). Deformation assisted phase transformation: An example from the sillimanite-in isograd, Eolus batholith, Needle Mountains, Colorado, USA. Terra Nova.

[13] Keller CB, Schoene B (2012). Statistical geochemistry reveals disruption in secular lithospheric evolution about 2.5 Gyr ago. Nature.

[14] Condie KC, Aster RC, van Hunen J (2016). A great thermal divergence in the mantle beginning 2.5 Ga: Geochemical constraints from greenstone basalts and komatiites. Geosci Front.

[15] Ganne J, Feng X (2017). Primary magmas and mantle temperatures through time. Geochem Geophys Geosyst.

[16] Keller CB, Schoene B (2018). Plate tectonics and continental basaltic geochemistry throughout Earth history. Earth Planet Sci Lett.

[17] Murphy JB, Nance RD (2003). Do supercontinents introvert or extrovert?: Sm-Nd isotope evidence. Geology.

[18] Cawood PA, Hawkesworth CJ (2014). Earth’s middle age. Geology.

[19] Liu C, Knoll AH, Hazen RM (2017). Geochemical and mineralogical evidence that Rodinian assembly was unique. Nat Commun.

[20] Dietz RS (1961). Continent and ocean basin evolution by spreading of the sea floor. Nature.

[21] Clift PD, Vannucchi P, Morgan JP (2009). Crustal redistribution, crust–mantle recycling and Phanerozoic evolution of the continental crust. Earth-Sci Rev.

[22] Jagoutz O, Kelemen PB (2015). Role of arc processes in the formation of continental crust. Annu Rev Earth Planet Sci.

[23] Ronov AB (1994). Phanerozoic transgressions and regressions on the continents: A quantitative approach based on areas flooded by the sea and areas of marine and continental deposition. Am J Sci.

[24] Bouvier A, Vervoort JD, Patchett PJ (2008). The Lu–Hf and Sm–Nd isotopic composition of CHUR: Constraints from unequilibrated chondrites and implications for the bulk composition of terrestrial planets. Earth Planet Sci Lett.

[25] Cherniak DJ (2003). Diffusion in zircon. Rev Mineral Geochem.

[26] Keller CB, Boehnke P, Schoene B (2017). Temporal variation in relative zircon abundance throughout Earth history. Geochem Perspect Lett.

[27] Cao W, Lee CTA, Lackey JS (2017). Episodic nature of continental arc activity since 750 Ma: A global compilation. Earth Planet Sci Lett.

[28] Keller CB, Schoene B, Barboni M, Samperton KM, Husson JM (2015). Volcanic–plutonic parity and the differentiation of the continental crust. Nature.

[29] Iizuka T, Yamaguchi T, Itano K, Hibiya Y, Suzuki K (2017). What Hf isotopes in zircon tell us about crust–mantle evolution. Lithos.

[30] Spencer CJ, Hawkesworth C, Cawood PA, Dhuime B (2013). Not all supercontinents are created equal: Gondwana-Rodinia case study. Geology.

[31] Gardiner NJ, Kirkland CL, Van kranendonk MJ (2016). The juvenile Hafnium isotope signal as a record of supercontinent cycles. Sci Rep.

[32] Hopkinson TN (2017). The identification and significance of pure sediment-derived granites. Earth Planet Sci Lett.

[33] Perfit MR, Gust DA, Bence AE, Arculus RJ, Taylor SR (1980). Chemical characteristics of island-arc basalts: Implications for mantle sources. Chem Geol.

[34] White WA (1973). Deep erosion by infracambrian ice sheets. Geol Soc Am Bull.

[35] Egholm DL, Nielsen SB, Pedersen VK, Lesemann JE (2009). Glacial effects limiting mountain height. Nature.

[36] Hoffman PF, Li ZX (2009). A palaeogeographic context for Neoproterozoic glaciation. Palaeogeogr Palaeoclimatol Palaeoecol.

[37] Rooney AD, Strauss JV, Brandon AD, Macdonald FA (2015). A Cryogenian chronology: Two long-lasting synchronous Neoproterozoic glaciations. Geology.

[38] Pu JP (2016). Dodging snowballs: Geochronology of the Gaskiers glaciation and the first appearance of the Ediacaran biota. Geology.

[39] Liu Y, Peltier WR (2013). Sea level variations during snowball Earth formation: 1. A preliminary analysis. J Geophys Res Solid Earth.

[40] Donnadieu Y, Fluteau F, Ramstein G, Ritz C (2003). Is there a conflict between the Neoproterozoic glacial deposits and the snowball Earth interpretation: An improved understanding with numerical modeling. Earth Planet Sci Lett.

[41] Hyde WT, Crowley TJ, Baum SK, Peltier WR (2000). Neoproterozoic ‘snowball Earth’ simulations with a coupled climate/ice-sheet model. Nature.

[42] PASSC (2001). Earth Impact Database.

[43] Geological Survey of Canada (1995).

[44] Pollard D, Kasting JF, Zugger ME (2017). Snowball Earth: Asynchronous coupling of sea-glacier flow with a global climate model. J Geophys Res Atmos.

[45] Hoffman PF (2017). Snowball Earth climate dynamics and Cryogenian geology-geobiology. Sci Adv.

[46] McMechan ME (2000). Vreeland diamictites–Neoproterozoic glaciogenic slope deposits, Rocky Mountains, northeast British Columbia. Bull Can Pet Geol.

[47] Hoffman PF (2017). Sedimentary depocenters on snowball Earth: Case studies from the Sturtian Chuos Formation in northern Namibia. Geosphere.

[48] Arnaud E, Halverson GP, Shields-Zhou G (2011).

[49] Rieu R, Allen PA, Etienne JL, Cozzi A, Wiechert U (2006). A Neoproterozoic glacially influenced basin margin succession and ‘atypical’ cap carbonate associated with bedrock palaeovalleys, Mirbat area, southern Oman. Basin Res.

[50] Bowring SA (2007). Geochronologic constraints on the chronostratigraphic framework of the Neoproterozoic Huqf Supergroup, Sultanate of Oman. Am J Sci.

[51] Rantakokko NE, Whitehouse MJ, Pease V, Windley BF (2014). Neoproterozoic evolution of the eastern Arabian basement based on a refined geochronology of the Marbat region, Sultanate of Oman. Geol Soc Spec Publ.

[52] Ross GM, Villeneuve ME (1997).

[53] Rose CV (2012). Constraints on the origin and relative timing of the Trezona δ13C anomaly below the end-Cryogenian glaciation. Earth Planet Sci Lett.

[54] Hallet B, Hunter L, Bogen J (1996). Rates of erosion and sediment evacuation by glaciers: A review of field data and their implications. Global Planet Change.

[55] Cowton T, Nienow P, Bartholomew I, Sole A, Mair D (2012). Rapid erosion beneath the Greenland ice sheet. Geology.

[56] Partin CA, Sadler PM (2016). Slow net sediment accumulation sets snowball Earth apart from all younger glacial episodes. Geology.

[57] Heizler MT, Harrison TM (1998). The thermal history of the New York basement determined from 40Ar/39Ar K-feldspar studies. J Geophys Res Solid Earth.

[58] Flowers RM, Bowring SA, Reiners PW (2006). Low long-term erosion rates and extreme continental stability documented by ancient (U-Th)/He dates. Geology.

[59] Karlstrom KE, Heizler M, Quigley MC (2010).

[60] DeLucia MS, Guenthner WR, Marshak S, Thomson SN, Ault AK (2017). Thermochronology links denudation of the Great Unconformity surface to the supercontinent cycle and snowball Earth. Geology.

[61] Molnár F, Watkinson DH, Jones PC (2001). Multiple hydrothermal processes in footwall units of the North Range, Sudbury Igneous Complex, Canada, and implications for the genesis of vein-type Cu-Ni-PGE deposits. Econ Geol.

[62] Gibson RL, WU R, Stevens G (1998). Thermal-metamorphic signature of an impact event in the Vredefort dome, South Africa. Geology.

[63] Brown M (2006). Duality of thermal regimes is the distinctive characteristic of plate tectonics since the Neoarchean. Geology.

[64] Harlow GE, Tsujimori T, Sorensen SS (2015). Jadeitites and plate tectonics. Annu Rev Earth Planet Sci.

[65] Sizova E, Gerya T, Brown M (2014). Contrasting styles of Phanerozoic and Precambrian continental collision. Gondwana Res.

[66] Palin RM, White RW (2016). Emergence of blueschists on Earth linked to secular changes in oceanic crust composition. Nat Geosci.

[67] Blackburn TJ (2012). An exhumation history of continents over billion-year time scales. Science.

[68] Chumakov NM (2010). Precambrian glaciations and associated biospheric events. Stratigr Geol Correl.

[69] Germs GJB, Gaucher C (2012). Nature and extent of a late Ediacaran (ca. 547 Ma) glacigenic erosion surface in southern Africa. S Afr J Geol.

[70] Chumakov NM (2011). Glacial deposits of the Bokson Group, East Sayan Mountains, Buryatian Republic, Russian Federation. Geol Soc Lon Memoirs.

[71] Egholm DL (2017). Formation of plateau landscapes on glaciated continental margins. Nat Geosci.

[72] Snyder FG (1947). The problem of the Lipalian interval. J Geol.

[73] White WA (1972). Deep erosion by continental ice sheets. Geol Soc Am Bull.

[74] Garrity CP, Soller DR (2009). http://pubs.usgs.gov/ds/424/.

[75] Licciardi JM, Clark PU, Jenson JW, Macayeal DR (1998). Deglaciation of a soft-bedded Laurentide ice sheet. Quat Sci Rev.

[76] Egyed L (1956). Determination of changes in the dimensions of the Earth from Palægeographical data. Nature.

[77] Armstrong RL (1969). Control of sea level relative to the continents. Nature.

[78] Hargraves RB (1976). Precambrian geologic history. Science.

[79] Lee CTA (2017). Deep mantle roots and continental emergence: Implications for whole-Earth elemental cycling, long-term climate, and the Cambrian explosion. Int Geol Rev.

[80] Whitehead JA (2017). Dimensions of continents and oceans–Water has carved a perfect cistern. Earth Planet Sci Lett.

[81] López-Gamundí OR, Buatois LA (2010).

[82] Herman F (2013). Worldwide acceleration of mountain erosion under a cooling climate. Nature.

[83] Gumsley AP, Chamberlain KR (2017). Timing and tempo of the great oxidation event. Proc Natl Acad Sci USA.

[84] Swanson-Hysell NL (2010). Cryogenian glaciation and the onset of carbon-isotope decoupling. Science.

[85] Childs OE (1985). Correlation of stratigraphic units of North America–COSUNA. AAPG Bull.

[86] Belousova E (2010). The growth of the continental crust: Constraints from zircon Hf-isotope data. Lithos.

[87] Dhuime B, Hawkesworth CJ, Cawood PA, Storey CD (2012). A change in the geodynamics of continental growth 3 billion years ago. Science.

[88] Spencer CJ (2014). Proterozoic onset of crustal reworking and collisional tectonics: Reappraisal of the zircon oxygen isotope record. Geology.

[89] Payne JL (2016). Strengths and limitations of zircon Lu-Hf and O isotopes in modelling crustal growth. Lithos.

[90] Zheng YF (2004). Zircon U-Pb and oxygen isotope evidence for a large-scale 18O depletion event in igneous rocks during the Neoproterozoic. Geochim Cosmochim Acta.

[91] MacLennan S (2018). The arc of the Snowball: U-Pb dates constrain the Islay anomaly and the initiation of the Sturtian glaciation. Geology.

[92] Morris JD, Leeman WP, Tera F (1990). The subducted component in island arc lavas: Constraints from Be isotopes and B–Be systematics. Nature.

[93] Crisp JA (1984). Rates of magma emplacement and volcanic output. J Volcanol Geotherm Res.

[94] Smith EI (1971). Determination of origin of small lunar and terrestrial craters by depth diameter ratio. J Geophys Res.

[95] Pike RJ (1974). Depth/diameter relations of fresh lunar craters: Revision from spacecraft data. Geophys Res Lett.

[96] Lambeck K, Smither C, Johnston P (1998). Sea-level change, glacial rebound and mantle viscosity for northern Europe. Geophys J Int.

[97] Laske G, Masters G, Ma Z, Paysanos M (2013). Update on CRUST1.0–A 1-degree global model of Earth’s crust. Geophys Res Abstr.

[98] Artemieva IM (2006). Global 1 x 1 thermal model TC1 for the continental lithosphere: Implications for lithosphere secular evolution. Tectonophysics.

[99] Larson RL (1991). Latest pulse of Earth: Evidence for a mid-Cretaceous superplume. Geology.

[100] Williams GD, Stelck CR, Caldwell WGE (1975). Speculations on the Cretaceous paleogeography of North America. The Cretaceous System in the Western Interior of North America: Geological Association of Canada Special Paper.

[101] Eriksen MC, Slingerland R (1990). Numerical simulations of tidal and wind-driven circulation in the Cretaceous Interior Seaway of North America. Geol Soc Am Bull.

[102] Hancock JM, Kauffman EG (1979). The great transgressions of the Late Cretaceous. J Geol Soc.

[103] Courtillot V, Olson P (2007). Mantle plumes link magnetic superchrons to phanerozoic mass depletion events. Earth Planet Sci Lett.

[104] Tarduno JA, Cottrell RD, Smirnov AV (2002). The Cretaceous superchron geodynamo: Observations near the tangent cylinder. Proc Natl Acad Sci USA.

[105] Zhang N, Zhong S (2011). Heat fluxes at the Earth’s surface and core–mantle boundary since Pangea formation and their implications for the geomagnetic superchrons. Earth Planet Sci Lett.

