# Supplementary material for: Neoproterozoic glacial origin of the Great Unconformity
Source: Proc Natl Acad Sci U S A. 2018 Dec 31;116(4):1136–45. doi: 10.1073/pnas.1804350116 (PMC6347685; doi:10.1073/pnas.1804350116)
Supplement: Supplementary File [file pnas.1804350116.sapp.pdf]

PNAS
www.pnas.org

Supplementary Information for

Neoproterozoic glacial origin of the Great Unconformity

C. Brenhin Keller, Jon M. Husson Ross N. Mitchell, William F. Bottke, Thomas M. Gernon
Patrick Boehnke, Elizabeth A. Bell, Nicholas L. Swanson-Hysell, and Shanan E. Peters

To whom correspondence should be addressed: C. Brenhin Keller
E-mail: cbkeller@dartmouth.edu

This PDF file includes:

Figs. S1 to S16
References for SI reference citations

www.pnas.org/cgi/doi/10.1073/pnas.1804350115

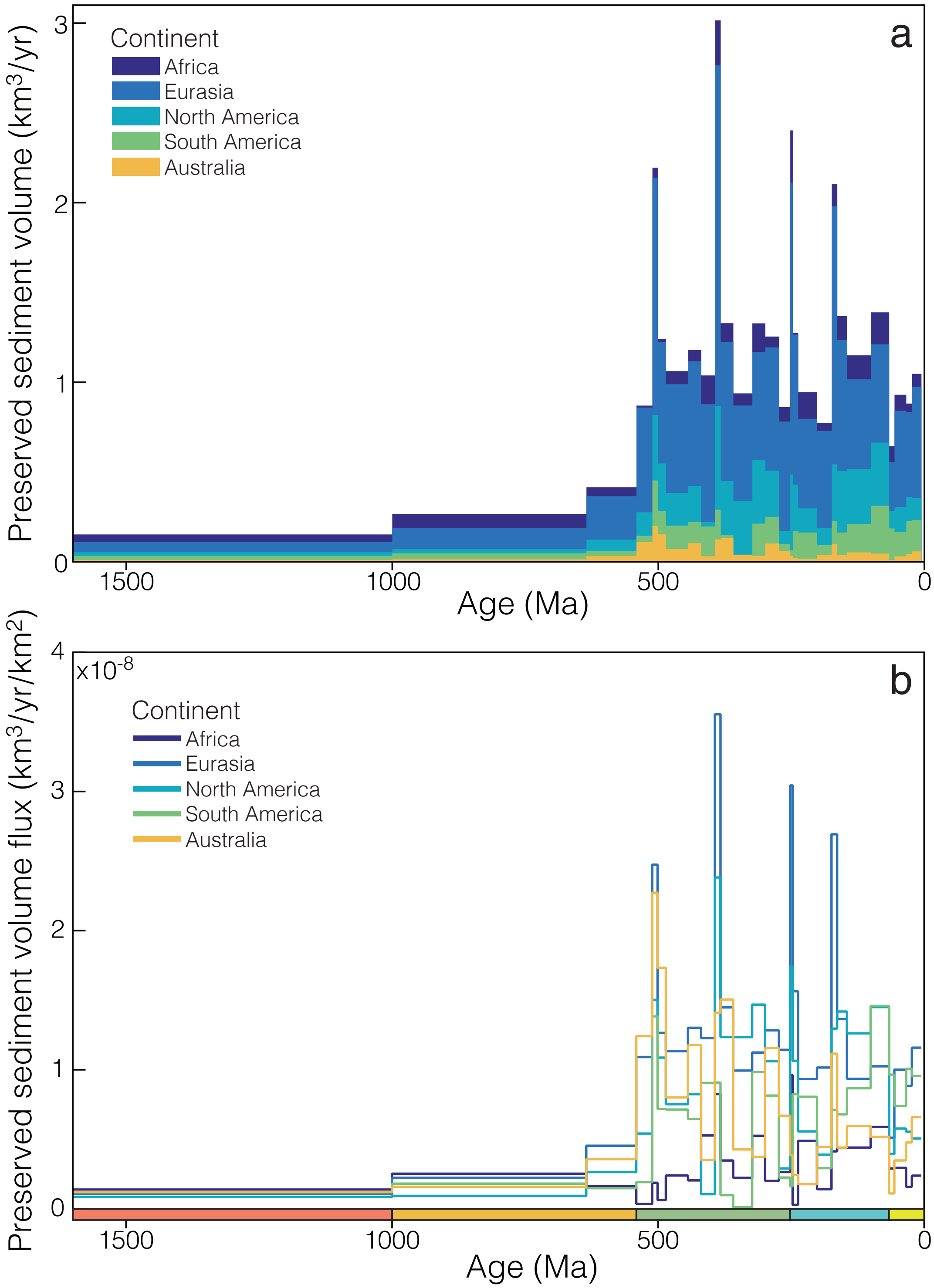

Fig. S1. The volumetric estimates of sedimentation on the continents compiled by Ronov and coauthors (1–15), as tabulated in Dataset S2. **(a)** Preserved sediment volumes for each continent, plotted cumulatively, in km^3 per year. **(b)** Volumetric flux, in km^3 per year per km^3 of continental surface area. All continents except Africa display a clear increase in preserved sediment volume and area-normalized sediment flux at the end of the Proterozoic.

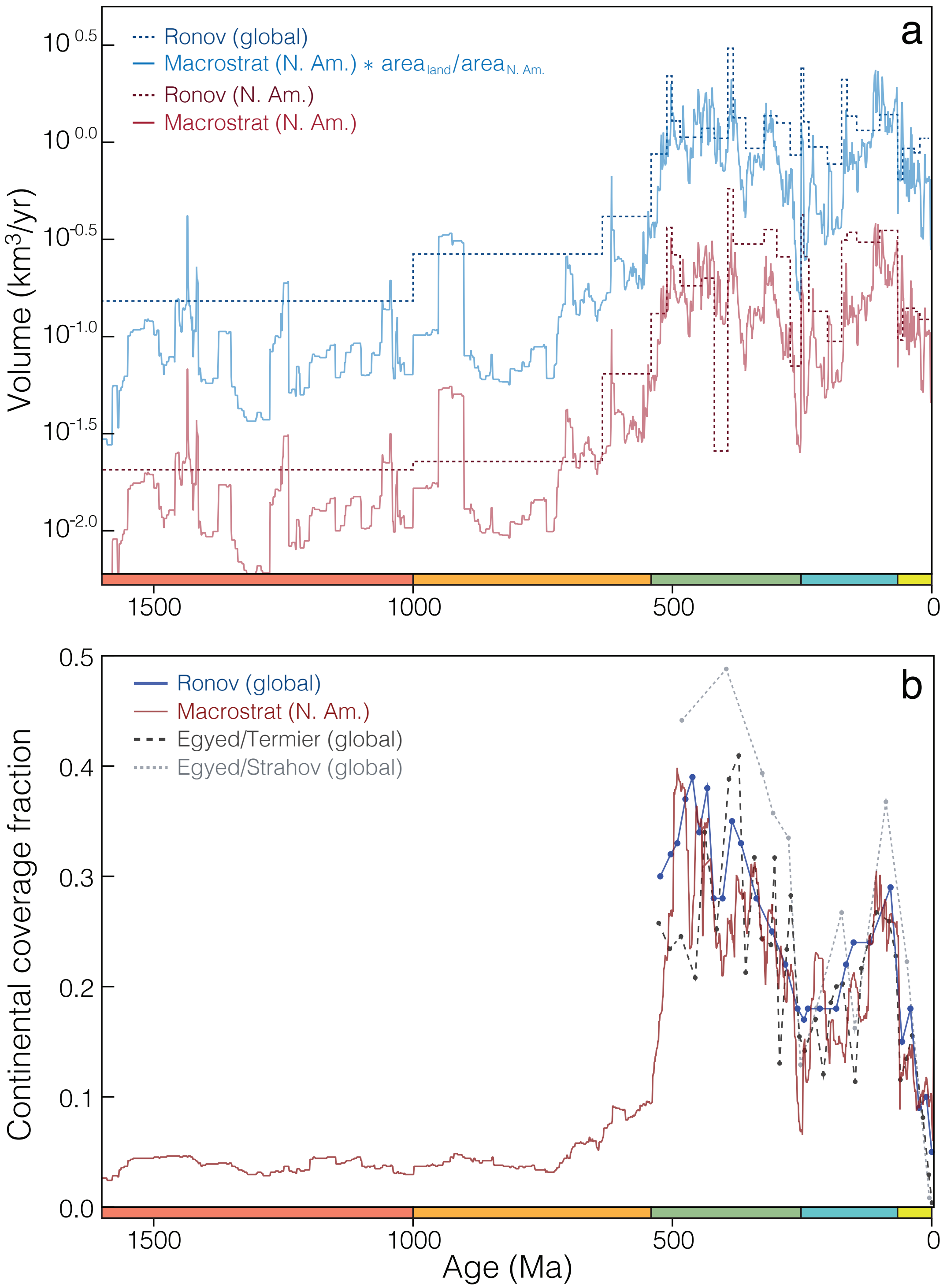

Fig. S2. Comparison of Macrostrat with global records. **(a)** Comparison of Ronov and Macrostrat sedimentary rock volume estimates. Blue: Ronov's (1) global record compared to a global scaling of the North American macrostrat record; that is, the Macrostrat sediment volume estimate for North America multiplied by the area of all continents divided by the area of North America (a factor of 6.1). Red: Ronov and coauthors' (1–15) estimate for preserved sediment volume on North America alone compared to the Macrostrat sediment volume estimate for North America. **(b)** The fraction of continental area covered by marine sediment, as estimated by Macrostrat and three global records: one compiled by Ronov (16), and two compiled by Egyed (17) on the basis of independent paleogeographic atlases, one due to Strahov (18) and the other to Termier & Termier (19).

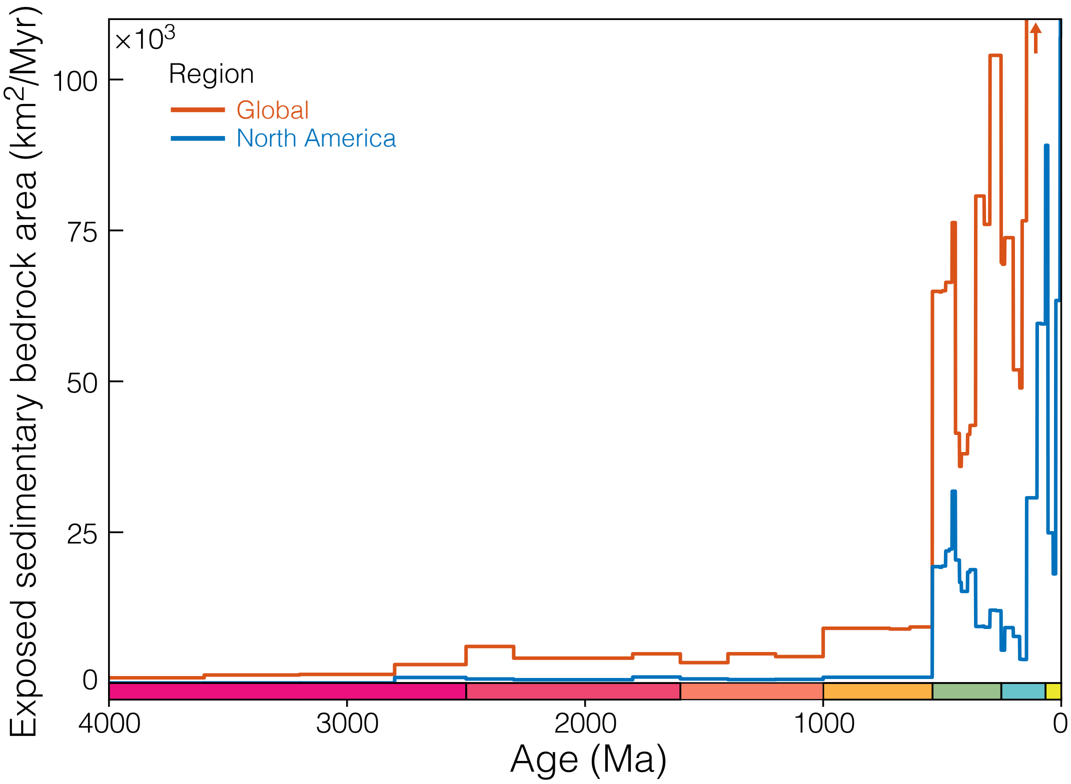

Fig. S3. Exposed surface area of sedimentary and metasedimentary rock as a function of depositional age, derived from the Geological Survey of Canada Generalized Geological Map of the World (20). In comparison to the sedimentary volume record of e.g. Fig. S1, this exposure-area record is significantly biased towards young Tertiary strata because it considers only the exposed uppermost strata of Earth's sedimentary shell. Nonetheless, a major increase in exposed area per unit depositional time is apparent at Proterozoic-Phanerozoic boundary.

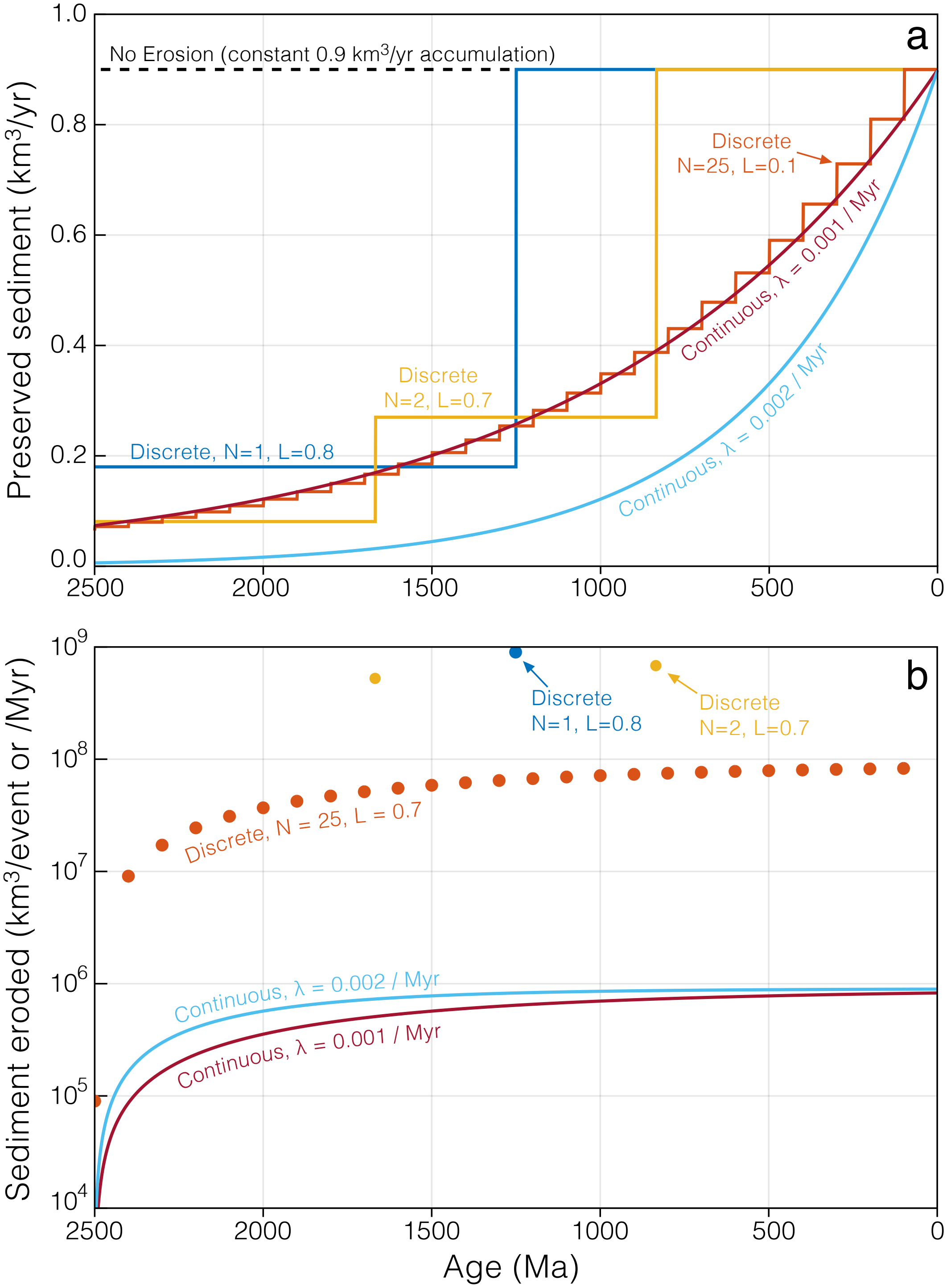

Fig. S4. Effects of continuous and discontinuous erosion in a model with constant 0.9 km^{3}y sediment input prior to erosion. Following Gregor (21), erosion is assumed to consume preexisting crust in proportion to its abundance. **(a)**: Preserved sediment volume per unit time for several imposed continuous and discontinuous erosion scenarios. **(b)**: Mass of sediment eroded per event or per unit time, for the same scenarios as in **(a)**. In the discontinuous erosion scenarios, erosion occurs during one or more discrete erosional events, producing step functions in preserved sediment volume. The strength of a given discrete erosional event is specified in terms of a loss factor: $L = 0.8$ specifies an erosional event in which 80% of all accumulated sediment present at the time of the event is eroded. The erosion rate in continuous erosion models, meanwhile, is determined by the decay constant λ, with units of $1/$Myr. Discontinuous models with N equally-spaced events of identical L converge towards the exponential form of continuous erosion as N becomes large. Note, for instance, the correspondence in preserved volume between the discrete model where 10% of the crust is lost every 100 Myr, and the continuous model with a $\lambda = 0.001/$Myr (i.e., 10%/100 Myr).

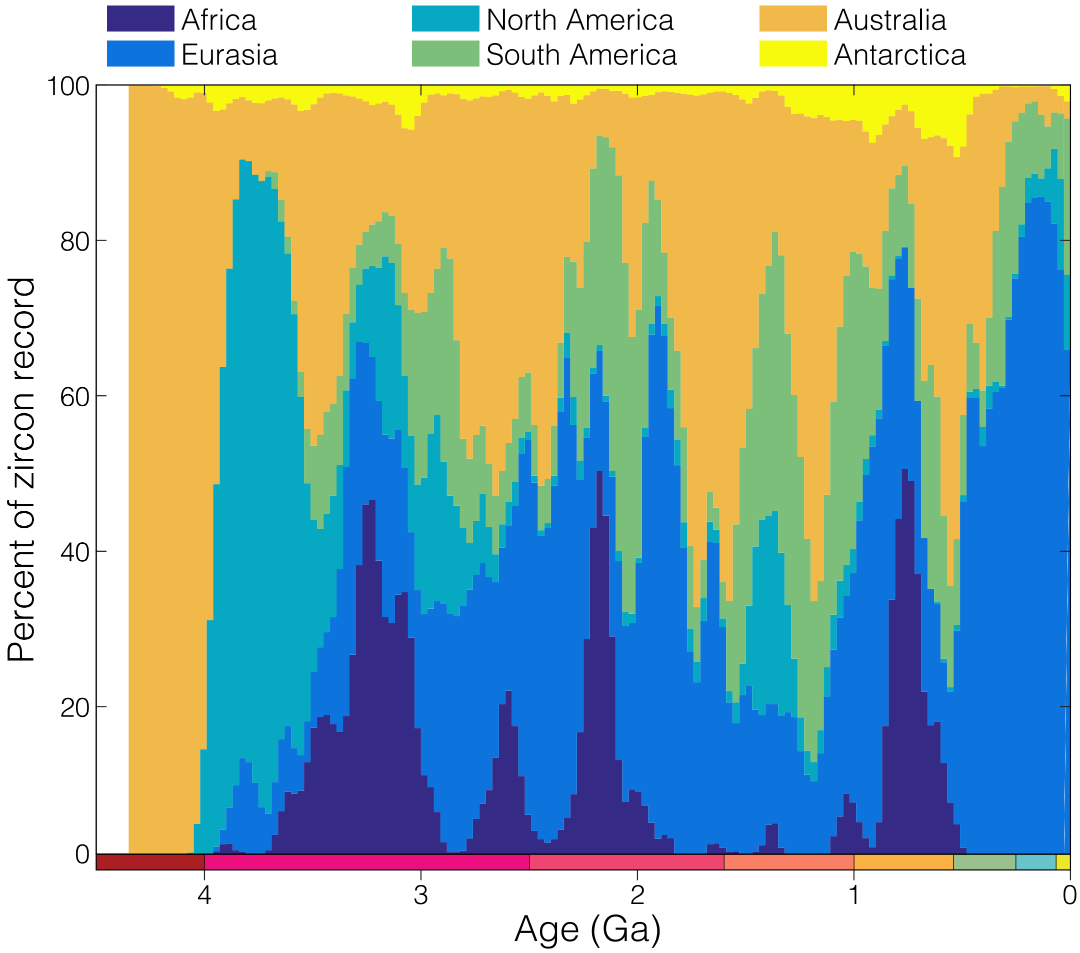

Fig. S5. Geographic distribution (by continent) of zircon Hf and O isotope analyses as a function of zircon U-Pb crystallization age in 200 Myr bins from 0-4.4 Ga. While relative abundances vary episodically in response to tectonic processes, no single continent dominates after 4 Ga.

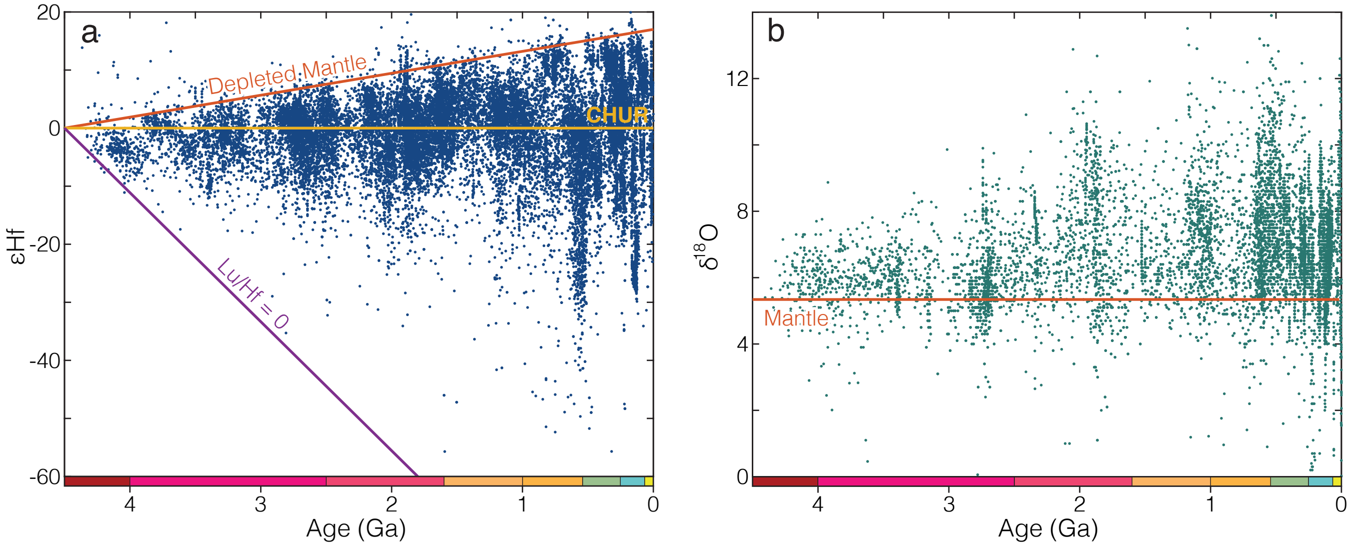

Fig. S6. All data points in the raw compiled zircon Hf and O **(b)** isotope datasets. The Hf isotope record **(a)** is constrained by the composition of the depleted mantle as a rough upper bound and the composition of a hypothetical preserved 4.5 Ga lithology with Lu/Hf = 0 as a strict lower bound. The zircon O isotope record is drawn to supra-mantle δ^{18}O by assimilation of siliciclastic sediment or silicate rock that has undergone low-temperature aqueous alteration.

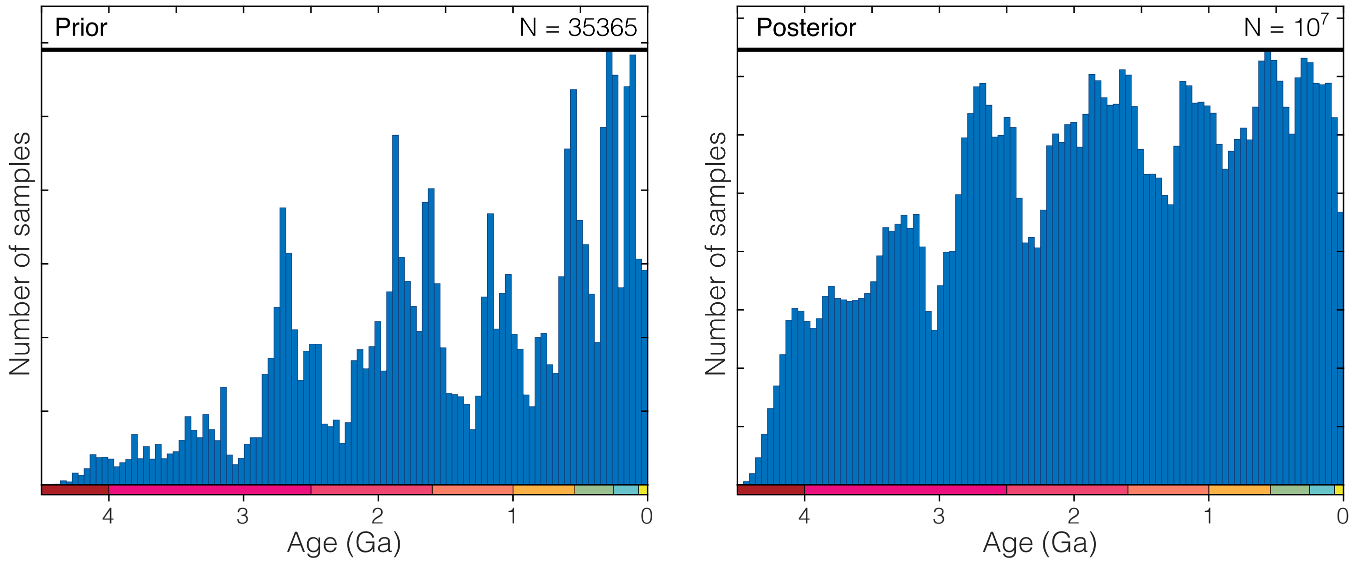

Fig. S7. Zircon age distributions of the raw dataset (prior) and the bootstrap-resampled dataset (posterior). Sample weighting results in a visibly more even posterior distribution.

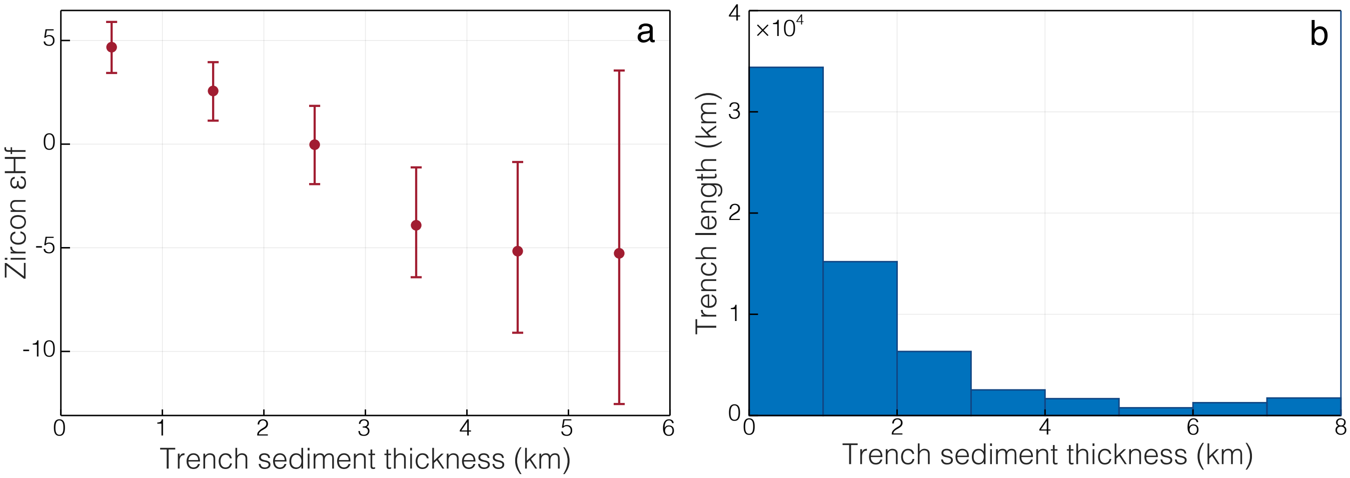

Fig. S8. The importance of sediment subduction to arc magma Hf isotope systematics is demonstrated by the clear variation in average zircon εHf as a function of present day trench sediment thickness for arc zircons younger than 100 Ma within 5 arc degrees of a trench of known sediment thickness following the sediment thickness maps of Heuret et al. (22) and the geospatially-resolved zircon Hf database of Bataille et al. (23). These data are resampled to accurately represent uncertainty in trench sediment thickness and binned in 1 km intervals. Extreme zircon Hf isotope compositions below -25 εHf are excluded, though this does not visibly influence the resulting trend.

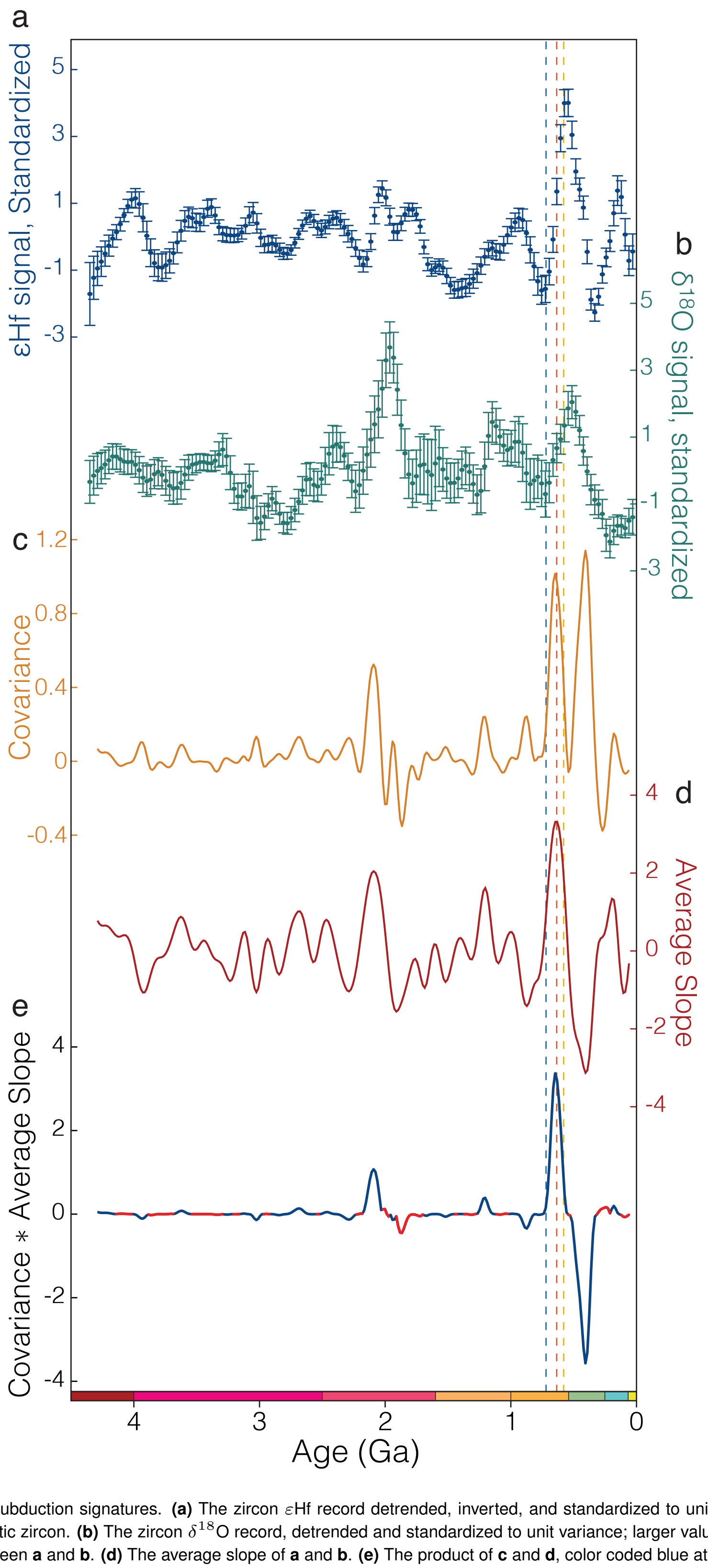

Fig. S9. Determination of sediment subduction signatures. **(a)** The zircon εHf record detrended, inverted, and standardized to unit variance; larger values indicate more recycling of old crust into new magmatic zircon. **(b)** The zircon δ^{18}O record, detrended and standardized to unit variance; larger values indicate more recycling of surficially altered crust. **(c)** The covariance between **a** and **b**. **(d)** The average slope of **a** and **b**. **(e)** The product of **c** and **d**, color coded blue at times of positive covariance and red at times of negative covariance. As explained in the Methods, sediment subduction events should appear as pairwise excursions with a positive peak for initiation and negative one for recovery.

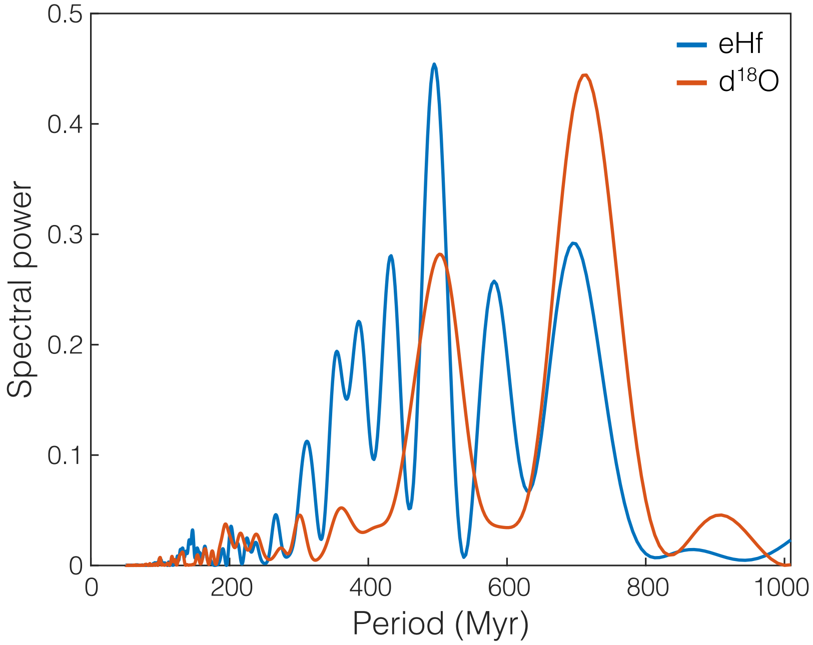

Fig. S10. Periodogram of the zircon Hf and O isotope records, highlighting substantial spectral power at 500-700 Myr periods consistent with tectonic cyclicity.

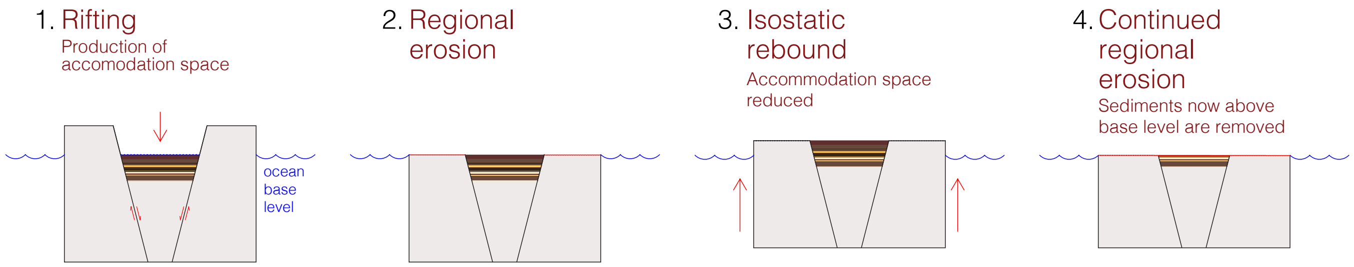

Fig. S11. Illustration of the competition between local tectonic subsidence and regional isostatic uplift under the influence of regional erosion. **1**: Accommodation space is produced by local rifting. **2**: Erosion removes regional uplands (horsts, in this case). **3**: Regional isostatic rebound. **4**: The tectonically-produced basin is now subject to destruction by continued regional erosion. The basin will survive if the rate of tectonic subsidence meets or exceeds the rate of regional erosion. If the rate of tectonic subsidence exactly matches the regional isostatic uplift from upland erosion, the basin will survive intact but with no new accumulation of sediments during the interval of regional erosion. In reality, isostatic rebound will occur continuously in concert with erosion; the two are separated here only to illustrate the underlying principle.

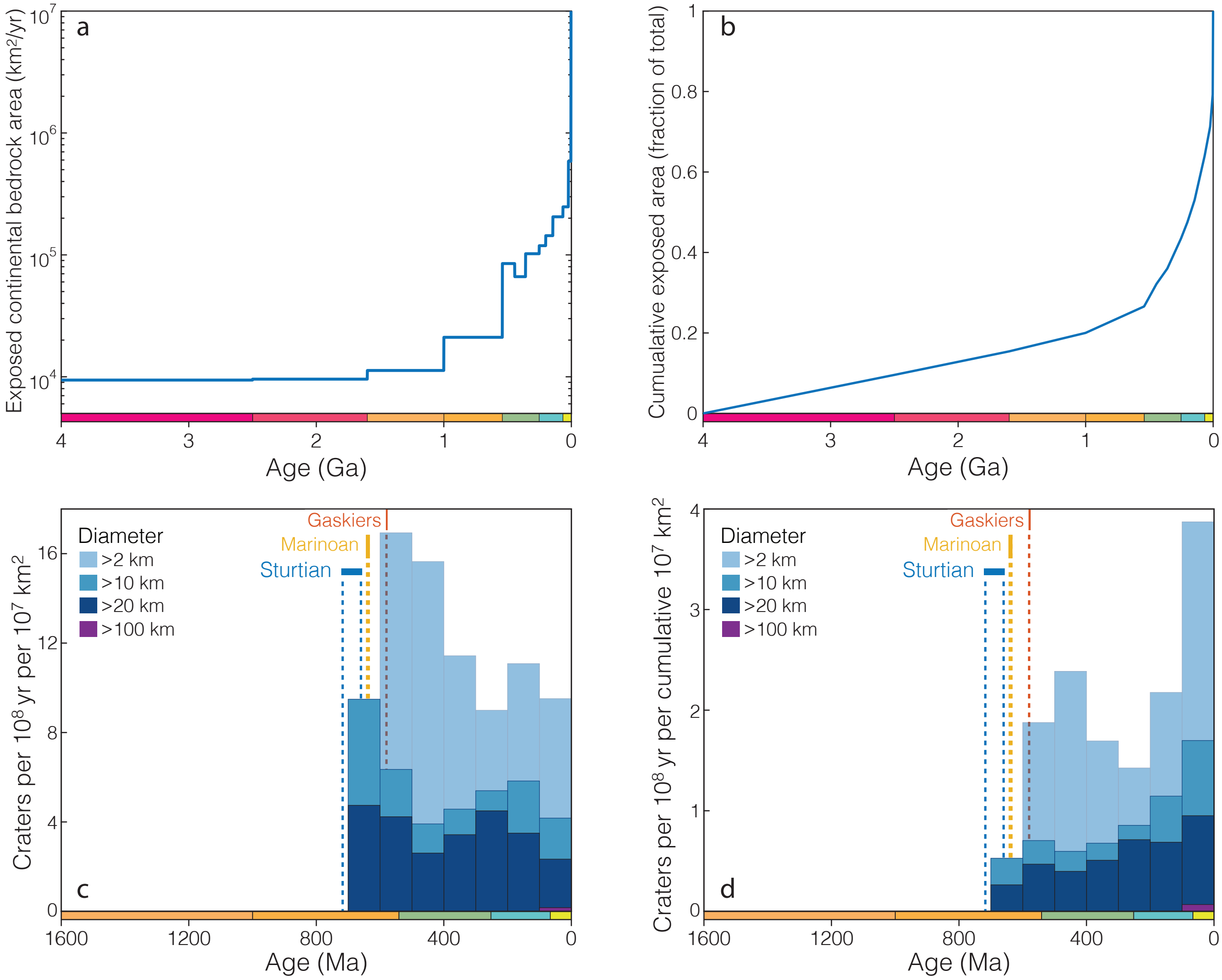

Fig. S12. Comparison of direct versus cumulative area normalizations of impact cratering rate. **(a)** Age distribution of continental bedrock exposed at the present day, derived from the Geological Survey of Canada Generalized Geological Map of the World (20). **(b)** Normalized cumulative age distribution of continental bedrock, obtained by integrating **a** from 4 Ga to time t. The cumulative total (1.0) is equal to the area of the continents, or $1.489 * 10^8$ km^2 **(c)** Impact cratering rate normalized by raw bedrock exposure from **a**; for sedimentary and volcanic bedrock, we know that this bedrock must have been exposed and susceptible for impact cratering at the time of deposition. **(d)** Impact cratering rate normalized by cumulative bedrock exposure from **b**.

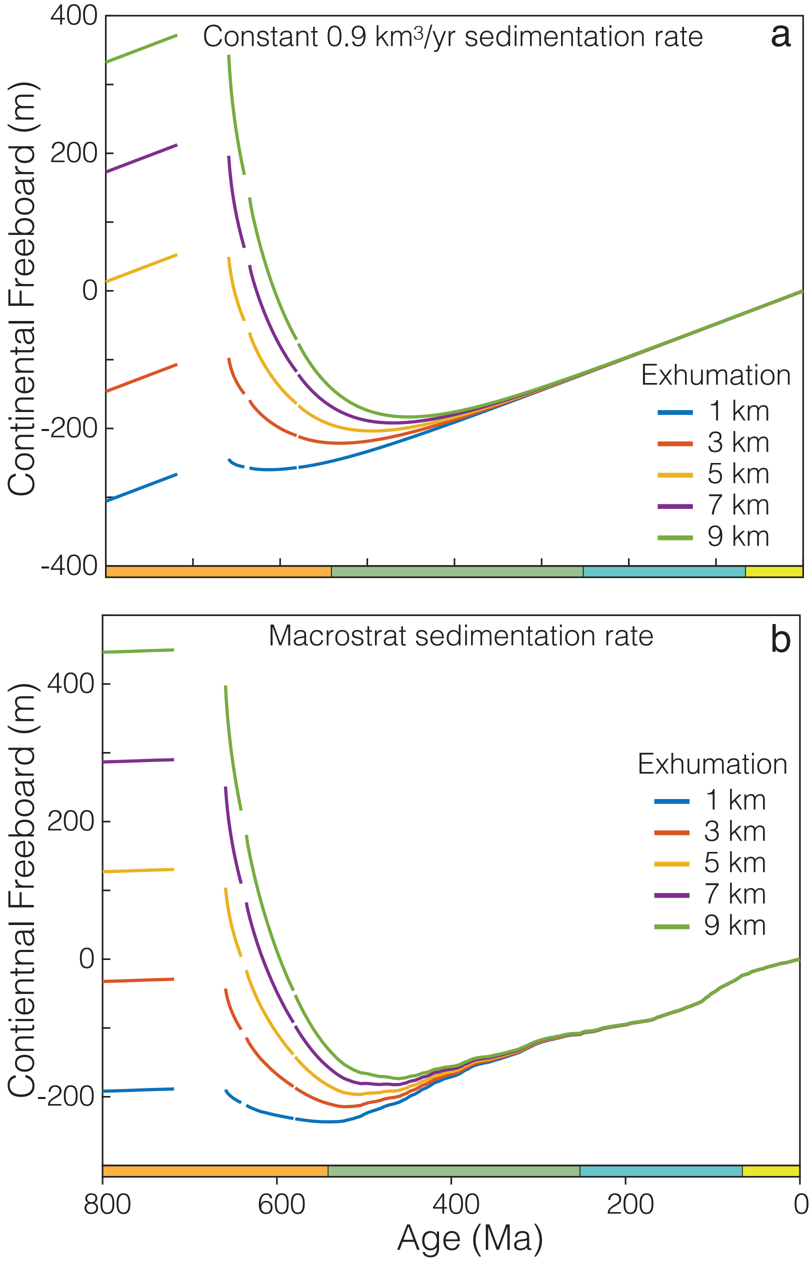

Fig. S13. The effect of variable magnitudes of Neoproterozoic erosion in an isostatic global sea level and continental coverage model. Here, the magnitude of glacial exhumation is varied between 1 and 9 km. Present-day sea level prior to the Neoproterozoic is reproduced with ∼4.5 km glacial erosion in the constant sedimentation model **(a)**, and ∼3.5 km in the variable sedimentation model **(b)**.

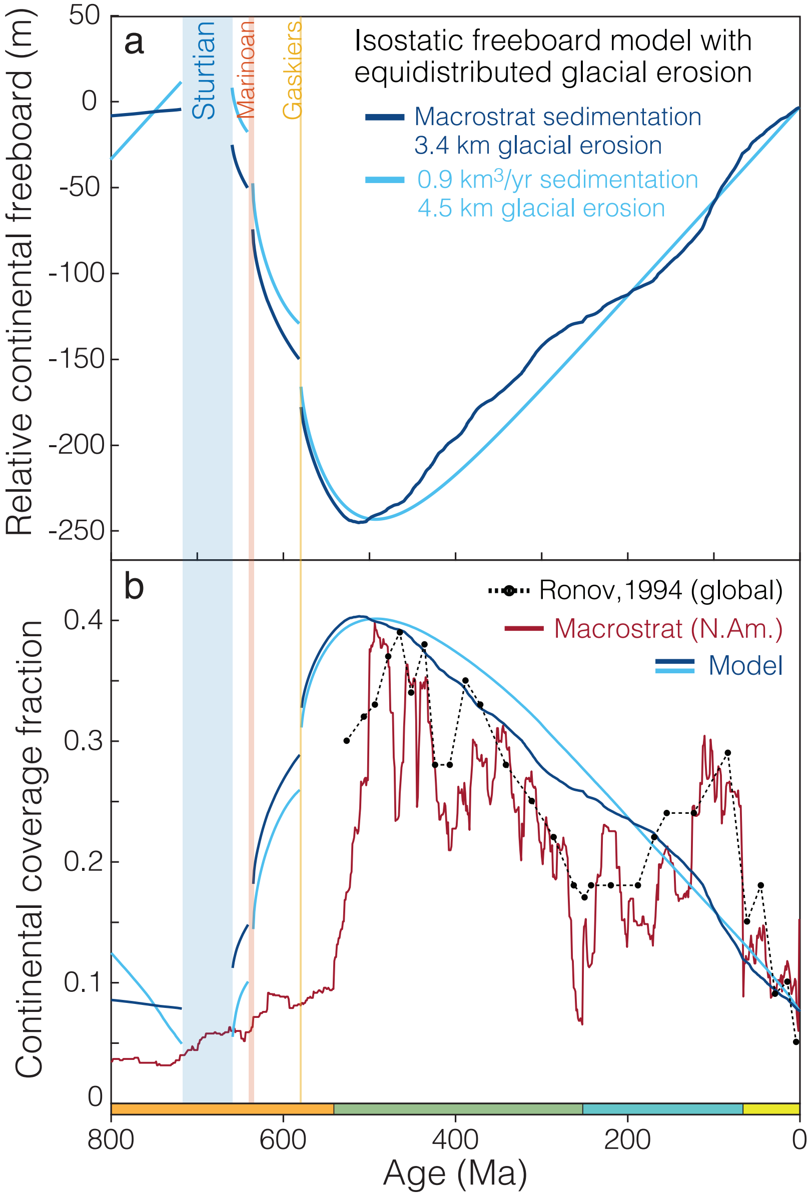

Fig. S14. Isostatic global sea level and continental coverage model, as in Fig. 4, but with glacial erosion equally distributed between the three glacial episodes. **(a)** Temporal evolution in average continental freeboard driven by erosion, subsequent thermal subsidence, and sediment accumulation. **(b)** Corresponding continental coverage fraction.

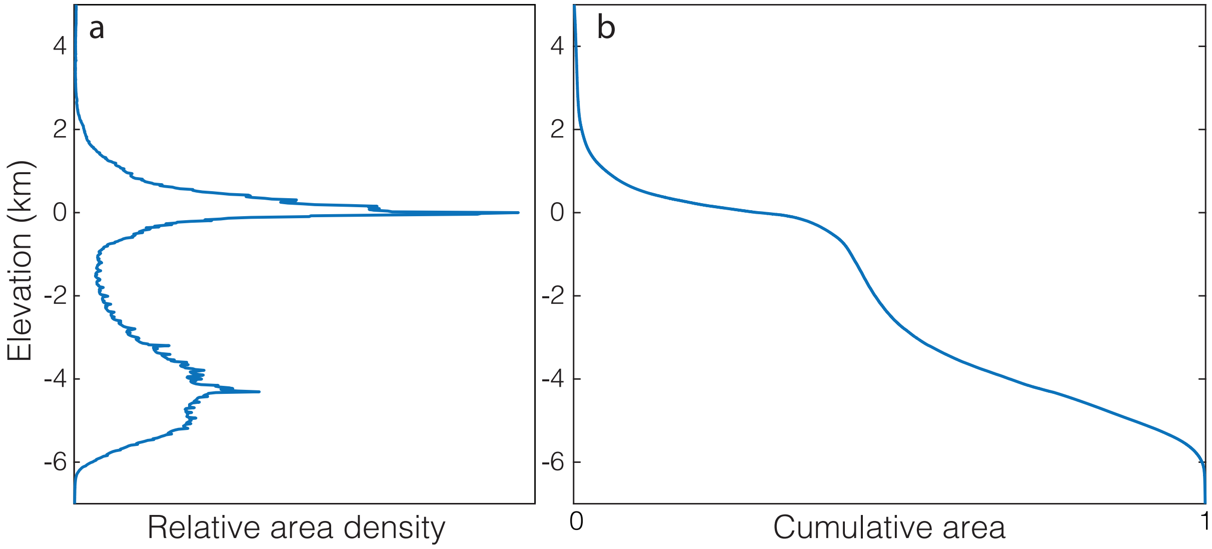

Fig. S15. Earth's present-day hypsometric curve, calculated from the ETOPO1 1 Arc-Minute Global Relief Model (24). The contrast between more dense oceanic crust and more buoyant continental crust is reflected in the clear bimodality in the distribution of elevations. **(a)** An estimate of Earth's elevation distribution based on the elevation of ETOPO1 grid cells. **(b)** Earth's cumulative elevation distribution, obtained by integrating **a** from high to low elevation. A cumulative area of 1.0 corresponds to the total surface area of the Earth.

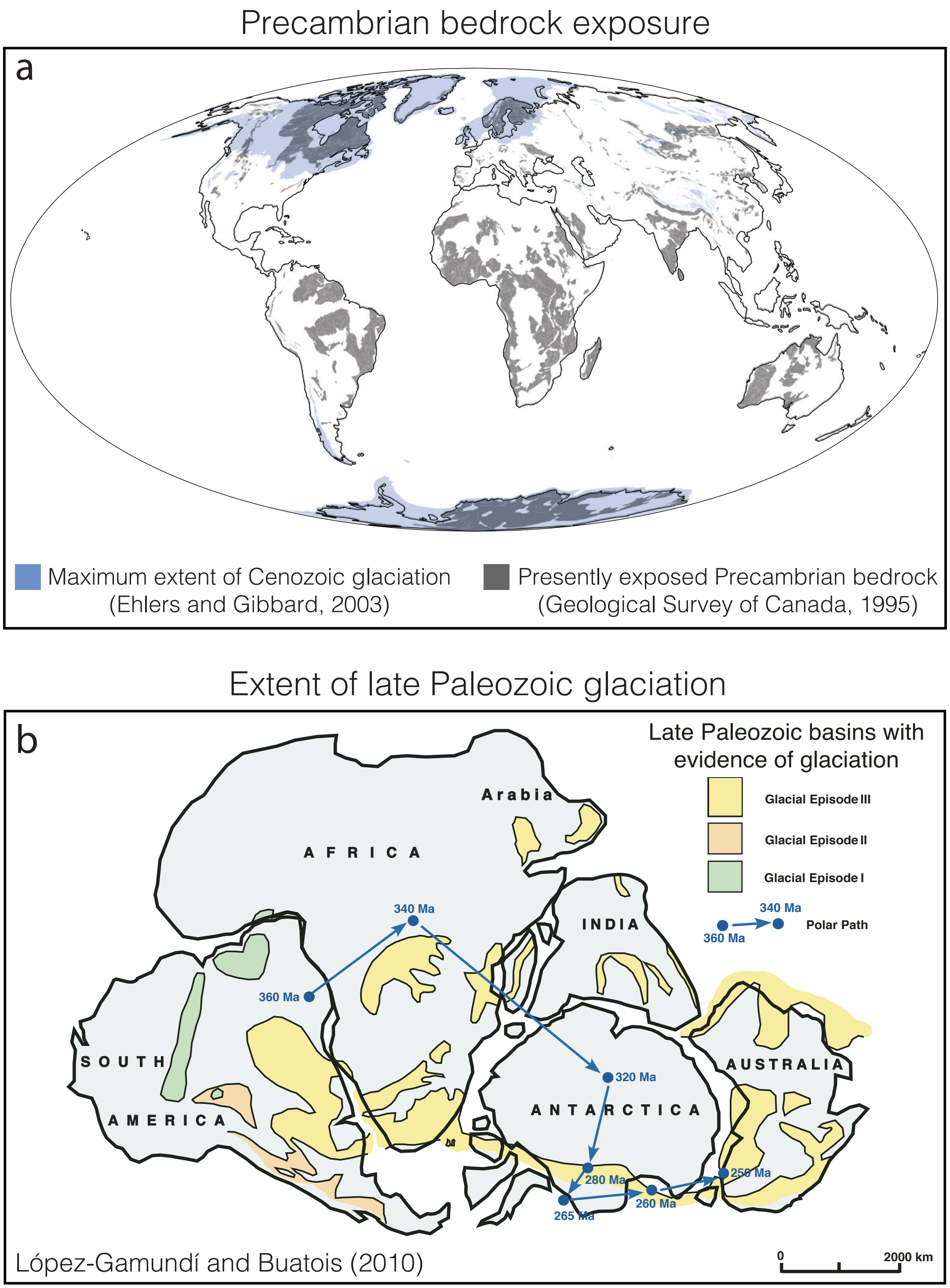

Fig. S16. Global correspondence between Precambrian basement exposures and Phanerozoic glaciation, as first noted by White (1972) (25). **(a)** Major exposures of Precambrian basement from the Geological Survey of Canada Generalized geological map of the world (20) overlain with the maximum extent of Cenozoic glaciation as compiled by Ehlers and Gibbard (26). **(b)** The extent of the Late Paleozoic Ice Age, modified with permission from ref. (27). Virtually all non-orogenic Precambrian basement exposures not covered by Pleistocene glaciation are of Gondwanan affinity, and were likely glaciated during the Late Paleozoic Ice Age. The correspondence between Gondwanan LPIA glaciation and basement exposure is less complete than that between Laurentide glaciation and the Canadian shield (Fig. 5), given that significant proportions of the glaciated area have been covered by Mesozoic and Cenozoic sediment in the intervening ∼250 Myr.

References

1. Ronov AB, Khain VE, Balukhovsky AN, Seslavinsky KB (1980) Quantitative analysis of Phanerozoic sedimentation. *Sedimentary Geology* 25(4):311–325.
2. Ronov AB, Khain VY, Seslavinskiy KB (1982) Lower and middle Riphean lithologic complexes of the world. *International Geology Review* 24(5):509–525.
3. Khain VY, Ronov AB, Seslavinskiy KB (1982) Upper Riphean lithologic complexes of the world. *International Geology Review* 24(9):993–1008.
4. Ronov AB, Khain VE, Seslavinskiy KB (1981) Vendian lithological complexes of the world. *Soviet Geology* (5):37–59.
5. Ronov AB, Seslavinskiy KB, Khain VY (1977) Cambrian lithologic associations of the world. *International Geology Review* 19(4):373–394.
6. Ronov AB, Khain VY, Seslavinskiy KB (1976) Ordovician lithologic associations of the world. *International Geology Review* 18(12):1395–1412.
7. Khain VY, Ronov AB, Seslavinskiy KB (1978) Silurian lithologic associations of the world. *International Geology Review* 20(3):249–268.
8. Ronov AB, Khain VE (1954) Devonian lithological formations of the world. *Soviet Geology* 41:46–76.
9. Ronov AB, Khain VE (1955) Carboniferous lithological formations of the world. *Soviet Geology* 48:92–117.
10. Ronov AB, Khain VE (1956) Permian lithological formations of the world. *Soviet Geology* 54:20–36.
11. Ronov AB, Khain VE (1961) Triassic lithological formations of the world. *Soviet Geology* (1):27–48.
12. Ronov AB, Khain VE (1962) Jurassic lithological formations of the world. *Soviet Geology* (1):9–34.
13. Khain VY, Ronov AB, Balukhovskiy AN (1976) Cretaceous lithologic associations of the world. *International Geology Review* 18(11):1269–1295.
14. Ronov AB, Khain VY, Balukhovskiy AN (1979) Paleogene lithologic associations of the continents. *International Geology Review* 21(4):415–446.
15. Khain VY, Ronov AB, Balukhovskiy AN (1981) Neogene lithologic associations of the continents. *International Geology Review* 23(4):426–454.
16. Ronov AB (1994) Phanerozoic Transgressions and Regressions on the Continents: a Quantitative Approach Based on Areas Flooded by the Sea and Areas of Marine and Continental Deposition. *American Journal of Science* 294(7):777–801.
17. Egyed L (1956) Determination of Changes in the Dimensions of the Earth from Palægeographical Data. *Nature* 178(4532):534–534.
18. Strahov NM (1948) *Outlines of Historical Geology*, in 2 volumes. (State Publishing Co. Geol. Lit., Moscow).
19. Termier H, Termier G (1953) *Histoire Géologique de la Biosphère*, La vie et les sédiments dans les géographies successives. (Masson et Cie, Éditeurs, Paris).
20. Geological Survey of Canada (1995) Generalized geological map of the world and linked databases, Technical Report 2915d.
21. Gregor B (1970) Denudation of the Continents. *Nature* 228(5268):273–275.
22. Heuret A, Conrad CP, Funiciello F (2012) Relation between subduction megathrust earthquakes, trench sediment thickness and upper plate strain. *Geophysical Research Letters* 39.
23. Bataille CP, Willis A, Yang X, Liu XM (2017) Continental igneous rock composition: A major control of past global chemical weathering. *Science Advances* 3(3):e1602183.
24. Amante C, Eakins BW (2009) *ETOPO1 1 arc-minute global relief model: procedures, data sources and analysis* (National Geophysical Data Center, NOAA).
25. White WA (1972) Deep Erosion by Continental Ice Sheets. 83:1037–1056.
26. Ehlers J, Gibbard PL (2003) Extent and chronology of glaciations. *Quaternary Science Reviews* 22(15-17):1561–1568.
27. López-Gamundí OR, Buatois LA (2010) Introduction: Late Paleozoic glacial events and postglacial transgressions in Gondwana in *Geological Society of America Special Paper 468*. (Geological Society of America), pp. v–viii.
